# Discovery of novel chemical reactions by deep generative recurrent neural network

**DOI:** 10.1038/s41598-021-81889-y

**Published:** 2021-02-04

**Authors:** William Bort, Igor I. Baskin, Timur Gimadiev, Artem Mukanov, Ramil Nugmanov, Pavel Sidorov, Gilles Marcou, Dragos Horvath, Olga Klimchuk, Timur Madzhidov, Alexandre Varnek

**Affiliations:** 1 Laboratory of Chemoinformatics, UMR 7140 CNRS, University of Strasbourg, 1, rue Blaise Pascal, 67000 Strasbourg, France; 2 Laboratory of Chemoinformatics and Molecular Modeling, Butlerov Institute of Chemistry, Kazan Federal University, Kremlyovskaya str. 18, 420008 Kazan, Russia; 3 Institute for Chemical Reaction Design and Discovery (WPI-ICReDD), Hokkaido University, Kita 21 Nishi 10, Kita-ku, Sapporo, 001-0021 Japan; 4 Department of Materials Science and Engineering, Technion – Israel Institute of Technology, 3200003 Haifa, Israel

**Keywords:** Cheminformatics, Chemical synthesis

## Abstract

The “creativity” of Artificial Intelligence (AI) in terms of generating de novo molecular structures opened a novel paradigm in compound design, weaknesses (stability & feasibility issues of such structures) notwithstanding. Here we show that “creative” AI may be as successfully taught to enumerate novel *chemical reactions* that are stoichiometrically coherent. Furthermore, when coupled to reaction space cartography, de novo reaction design may be focused on the desired reaction class. A sequence-to-sequence autoencoder with bidirectional Long Short-Term Memory layers was trained on on-purpose developed “SMILES/CGR” strings, encoding reactions of the USPTO database. The autoencoder latent space was visualized on a generative topographic map. Novel latent space points were sampled around a map area populated by Suzuki reactions and decoded to corresponding reactions. These can be critically analyzed by the expert, cleaned of irrelevant functional groups and eventually experimentally attempted, herewith enlarging the synthetic purpose of popular synthetic pathways.

## Introduction

The discovery of new organic reactions has always been in the focus of synthetic organic chemistry. Each new reaction enriches the arsenal of synthetic tools and opens new horizons in the development and optimization of new drugs and materials. Such reactions are often given the names of their discoverers, which is the highest recognition of their contribution to organic chemistry. Most of the new reactions have been discovered by plain luck, and it has been up to the chemists to notice the discovery and apply their “chemical intuition” to study it in detail^1^. The beginning of a systematic approach to the search for new reactions was laid in 1967 by Balaban, who applied the graph theory for systematical enumeration of pericyclic reactions proceeding through a 6-membered transition state^2^. In the 1970s, these studies were significantly expanded by Hendrickson^3^, Arens^4–6^, Zefirov, and Tratch^7,8^ who considered various formal schemes describing bonds redistribution for different types of pericyclic reactions. Another approach implemented in the IGOR^1,9^ and IGOR2^10^ programs concerned the algebraic model of constitutional chemistry developed by Dugundji and Ugi^11^. This approach supports the hierarchical representation of organic reactions and deals explicitly with heteroatoms and charges, keeps track of rings in molecules^10^. Its application led to the discovery of previously unknown reactions: the thermal decomposition of α-formyl-oxy ketones^1,9^, and the formation of a cage molecule from N-methoxycarbonyl homopyrrole and tropone^10^. Then, an alternative method based on the generation of the complete sets of non-isomorphic spanning subgraphs of a given graph was suggested. With the help of this approach, new carbene reaction^12^ and two new elimination reactions leading to the formation of synthetically important dienes^13^ were discovered. The formal-logical approach to organic reactions^7^ implemented in the SYMBEQ^14^ and ARGENT^15,16^ software was used to discover substituted furans^14^.

Despite great expectations, no significant progress in computer-aided reaction design was achieved; approaches, algorithms, and software tools reported so far have not found any widespread popularity among organic chemists. The work with those tools required both extensive knowledge in synthetic organic chemistry and a well-developed intuition to turn abstract schemes of bonds redistribution into specific chemical reactions with particular reagents, catalysts, and experimental conditions. This explains why all reactions computationally discovered so far were relatively simple (mainly thermal pericyclic reactions). We believe that real progress in the discovery of new chemical reactions can be achieved by deep learning from big data^17^. Recently, Segler et al. reported a chemical synthesis planning system based on deep neural networks and symbolic AI trained on a big collection of known synthetic reactions^18^. This tool, however, implements automatic extraction of transformation rules (“templates”) from known chemical reactions and therefore, in principle, cannot “suggest” not yet seen transformations. Several template-free techniques based on recurrent neural networks and transformers were successfully implemented. They operate in sequence-to-sequence translation mode^19^, in which SMILES of products were directly predicted from SMILES of reactants^20,21^ and vice versa^22–24^. Interesting chemistry knowledge driven approaches aiming to predict organic reactions outcomes from given reactants were proposed by Coley et al.^25^ and in Baldi’s group^26, 27^. Although, discovery of new chemical transformations cannot be excluded, this is not an objective of such type of calculations. To our knowledge, no new types of chemical reactions resulted from the “reactants-to-products” models were reported in the literature so far.

Generative models based on recurrent deep neural networks were successfully used to generate novel chemical structures^28–37^. Recently, we have demonstrated that the structures of molecules possessing desirable properties could be generated using a combination of autoencoder with Generative Topographic Map built on the latent vectors^26^. In order to apply this approach to chemical reactions, they must be encoded by SMILES strings. However, conventional reaction SMILES can hardly be used because: (i) they are much longer, and (ii) atom-to-atom mapping (AAM) needed for reaction center identification, adds a further layer of complexity. The autoencoder would have to learn not only semantics and syntax of SMILES but also the AAM rules.

**Figure 1:**
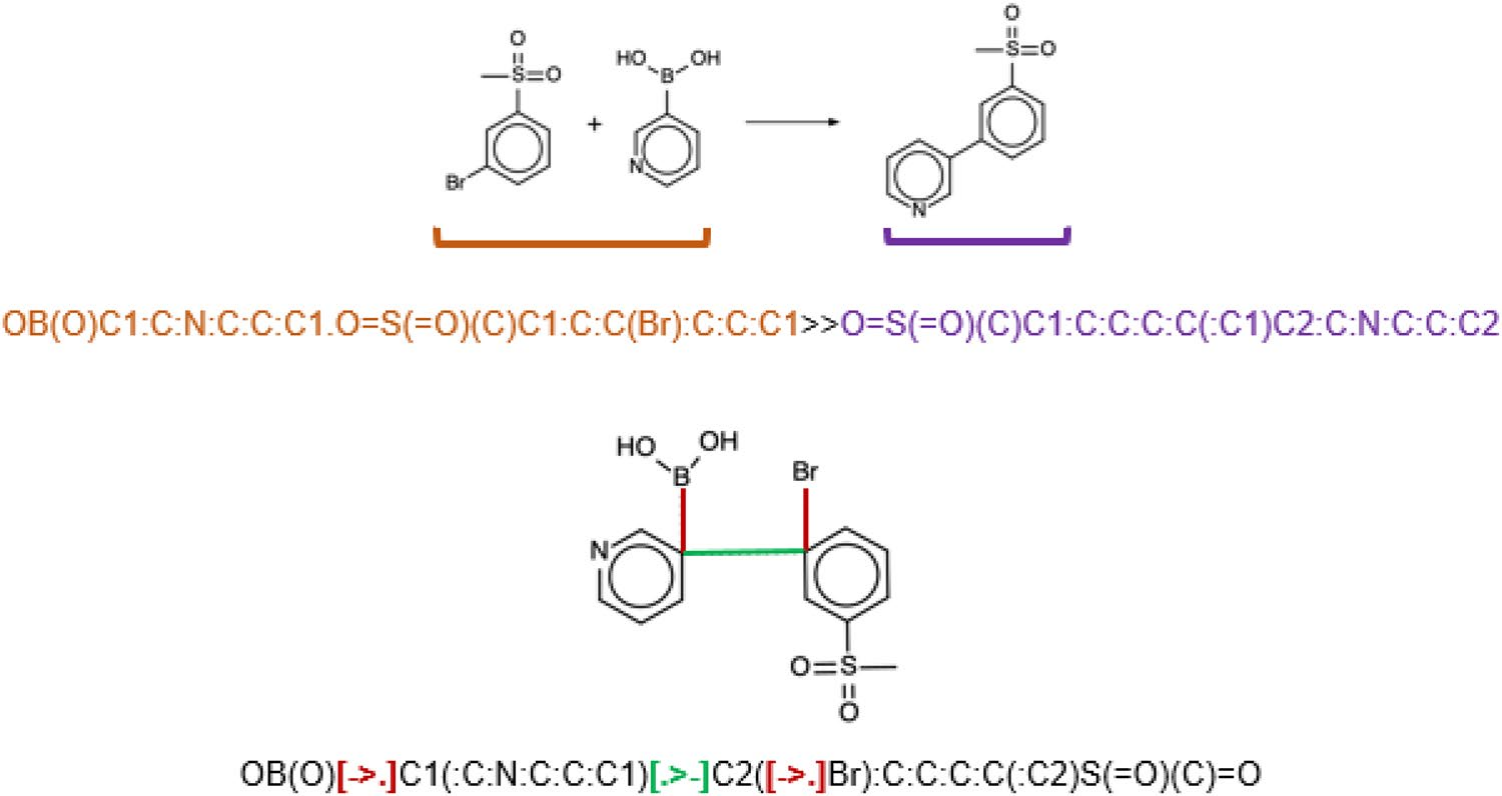
An example of Suzuki coupling reaction (*top*) and its condensed graph (CGR, *bottom*). Reaction SMILES and SMILES/CGR are given underneath. The reaction SMILES features reactants (in orange), and products (in purple). Atom-to-atom mapping is not provided. In the SMILES/CGR broken single bonds are encoded as [- > .] (in red), while the created C–C bond is [. > -] (in green). The colon (:) represents aromatic bonds. See Supporting Information for the details.

Earlier, we showed that in silico chemical reaction handling can be significantly simplified by the Condensed Graph of Reaction (CGR) approach^38^, in which the structures of reactants and products are merged into a single r graph (Fig. 1). The CGR edges correspond either to standard chemical bonds or to “dynamic” bonds describing transformations. In such a way, one can consider a CGR as a pseudomolecule for which some types of molecular descriptors can easily be computed followed by their application in data analysis and statistical modeling tasks^39^. Thus, this approach was successfully applied to similarity searching in reaction databases^38,40^, building quantitative structure–reactivity models^41–44^, assessment of tautomer distributions^45,46^, prediction of activity cliffs^47^, classification of enzymatic transformations^48^, prediction of reaction conditions^49,50^, etc. Here, for the first time, we introduce dedicated SMILES strings encoding CGRs (SMILES/CGR), see their detailed description in Supporting Information. Moreover, the CGR (and, hence, SMILES/CGR) contains information about the reaction center and its close neighborhood^51^.

Basically, CGRs are nothing but “molecules” with “exotic” bond orders for the changing bonds—thus, let us “teach” de novo molecular design tools on how to generate new reactions! Following a workflow recently used for the generation of novel molecular structures potentially possessing desirable biological activity^30^, we have chosen to focus here on the generation of “Suzuki-like” putative chemical transformations. The Suzuki coupling reaction was chosen because this reaction is widely used in organic synthesis, and, therefore, its new variants implying different leaving groups and reaction centers could be of interest for synthetic chemists. From the technical point of view, Suzuki reactions constitute a sizeable part of the USPTO database which assures satisfactory knowledge extraction upon the model training. Reaction center of Suzuki reaction can be represented by a SMILES string BC.**QL** >  > B.C**Q**.**L** (where **Q** = C, N, O, S, Si and **L** is a leaving group). In our simulation we expect that AI may suggest realistic and unseen combinations of **Q** and **L**.

**Figure 2:**
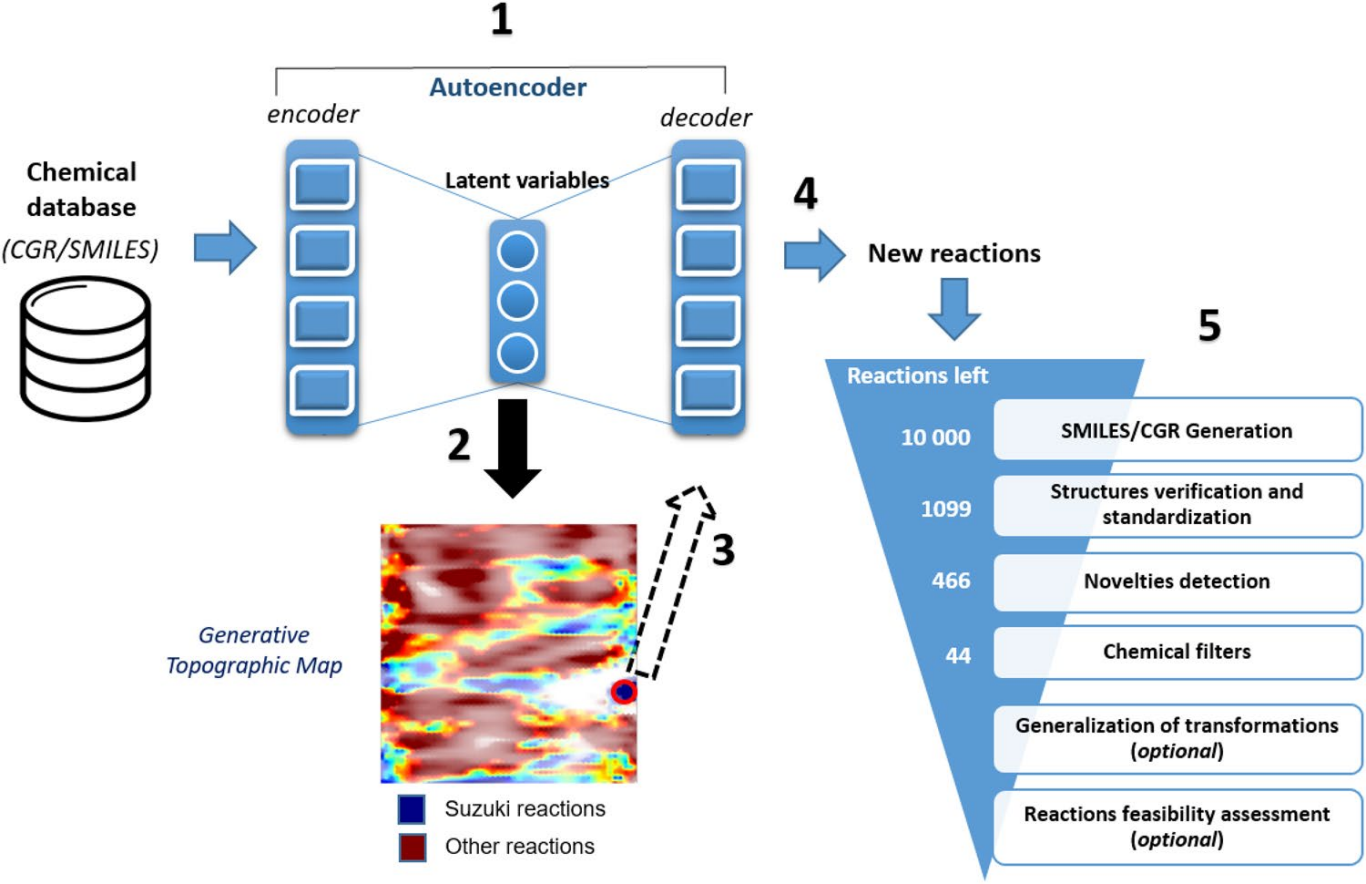
Modeling workflow for generation of new reactions consists of five main steps: (1) training sequence-to-sequence autoencoder on the USPTO database of chemical reactions; (2) building of Generative Topographic Map (GTM) using the autoencoder latent variables and preparation of GTM class landscape; (3) selecting on GTM a zone populated to Suzuki coupling reactions and identification of related autoencoder latent vectors; (4) sampling from the autoencoder latent space and generation of new reactions; and, (5) post-processing step. On the Generative Topographic Map, larger transparency levels correspond to lower density. The color code renders the (binary: Suzuki *vs* Other) reaction class distribution. Thus, zones in dark blue are exclusively populated by Suzuki reactions, zones in dark red are exclusively populated by other types of reactions; while intermediate colors correspond to reaction space areas hosting both categories, in various ratios. The red circle indicates the zone from which virtual Suzuki reactions were sampled.

A sequence-to-sequence neural network with Bidirectional Long Short-Term Memory^52^ layers trained on SMILES/CGR achieved the ability to convert SMILES/CGR to their latent vectors (“encode”) and back (“decode”). Generative Topographic Mapping (GTM) was used to visualize the latent space in 2D and to detect a cluster mostly populated with Suzuki reactions (Fig. 2). Then, virtual chemical reactions were generated by sampling the targeted zone followed by the decoding of associated latent vectors to SMILES/CGR. Notice that visualization is not strictly required for clusters identification, but may significantly help to choose a cluster from which the sampling is performed.

## Results

### Reaction sampling from generative topographic map

A set of 2 424 306 reactions, extracted and curated from the USPTO database^53^, was rendered as CGRs and then as SMILES/CGR strings used to feed the autoencoder. The latter was trained on some 2 million reactions and validated on 450 thousand reactions. The reconstruction rate (a ratio of correctly reconstructed SMILES/CGR) was 98.4% and 97.8% at the training and validation stage, respectively. This is slightly less than reconstruction rates of plain molecular SMILES by state-of-the-art encoders/decoders, but it can be explained by larger complexity and length of SMILES/CGR and an additional source of error: the errors of atom-to-atom mapping in some entries. SMILES/CGR is intrinsically more difficult to learn, with dynamical bonds, dynamical atoms and formal coordination numbers exceeding atomic valency representing novel degrees of freedom in the syntax. Unbalanced or erroneous entries may pass the standardization protocols and thus negatively impact generated SMILES/CGR quality.Nevertheless, reconstruction rates are robust and although LSTM has relatively short memory compared to some other neural networks architectures like transformers and, therefore, may fail to learn relatively complex structural motifs, the bidirectional LSTM used in our work seems to perform acceptably well.

The latent vectors for 100 000 randomly selected reactions were used to construct a Generative Topographic Map (GTM) using in-house software^54^. Then the entire USPTO database was projected onto the map, on which several zones predominantly populated by Suzuki reactions were identified, as shown in Fig. 2.

Random latent vectors were sampled from one of these zones with the highest relative population of Suzuki reactions. As expected, the sampling procedure led to virtual transformations of a similar type. Finally, 10,000 text strings have been generated, followed by their analysis using a complex post-processing protocol (Fig. 2). At the structures verification and standardization stage, the CGRtools.v3 tool was used to discard invalid SMILES/CGR and to perform valence and aromaticity check. This reduced the dataset to 1099 reactions (some 11% of generated text strings) in which structures of reactants and products were correct. This value is similar to that (15–20%) observed for the SMILES strings in our previous studies devoted to generation of individual molecules. Clearly, not every latent space vector corresponds to a valid structure. However, since invalid SMILES/CGR can be discarded algorithmically, they are not a liability but a manageable consequence of exploratory sampling.

Also, the USPTO reactions are unevenly distributed in terms of types. Deep learning typically focuses on dense clusters allowing it to extensively capture their associated syntactic rules. The realibility of different combinations of leaving group **L** and a coupling partner **Q** in the reaction center BC.**QL** >  > B.C**Q**.**L** suggested by AI depends not only on the training set size but also on its diversity, i.e., on the presence in the training set related examples. The USPTO dataset contains very few reactions with **Q** = O, S and Si which may explain the relatively high rate of invalid SMILES/CGR strings.

### Reaction novelty analysis

The main interest of in silico reaction generation is the proposal of novel reactions that a human mind would not spontaneously think of. However, unlike individual compounds, where novelties can be identified as unique scaffolds or particular structural motifs^30^, the definition of reaction novelty was not discussed in the literature. The most descriptive part is the reaction center (***RC***)^55^, i.e. atoms and bonds directly involved in the transformation. Thus, we consider two levels of reaction novelty: (i) the reaction center is unknown (not present in the training set); (ii) reaction center is known, but its closest neighborhood (1^st^ atoms and bonds near the RC, ***RC + 1***) is new. The latter can be extended to a more distant neighborhood (*n* atoms and bonds away, ***RC + n***), but in this work, we only focus on the reaction center and the closest neighbors. To decide whether a reaction is novel, these substructural reaction motifs are encoded by a hashing function as reaction signatures and are compared to all signatures extracted from the initial dataset.

Among 1099 reactions selected using the post-processing workflow (Fig. 2), 436 contain new reaction center ***RC*** and 30 reactions are novel at first neighborhood level ***RC + 1***. Some generated reactions have two or more distinct reaction centers, i.e. represent multistep transformations. Note that “novelty” defined as the absence of reaction center from the training set data is per se meaningful, as an illustration of the “creativity” of this Artificial Intelligence, i.e. its ability to generate original reaction centers which can be submitted for empirical feasibility assessment to human experts. Unfortunately, “novelty” as the absence of reaction center from both the training set and public reaction databases is not easy to interpret, for it may both mean that (a) such reactions were tried, but failed and thus were not published or (b) reactions were never explored, thus represent a real asset of innovation. The choice not to publish failed reactions is a major drawback in training reactivity models^44^.

### Reactions curation and generalisation

A close look at the generated reactions reveals several serious drawbacks: (i) unbalanced reaction equations, (ii) presence of likely unstable groups (e.g., R_3_S(=O)H and R–PH(=O)–OR’), and, (iii) transformations which require harsh reaction conditions (e.g., breaking of a C–C bond), or kinetically unfavorable reactions (e.g., cleavage of a leaving group with carbon at attachment point). Some reactions can be corrected or discarded using some heuristic rules (“Chemical Filters”).

**Figure 3:**
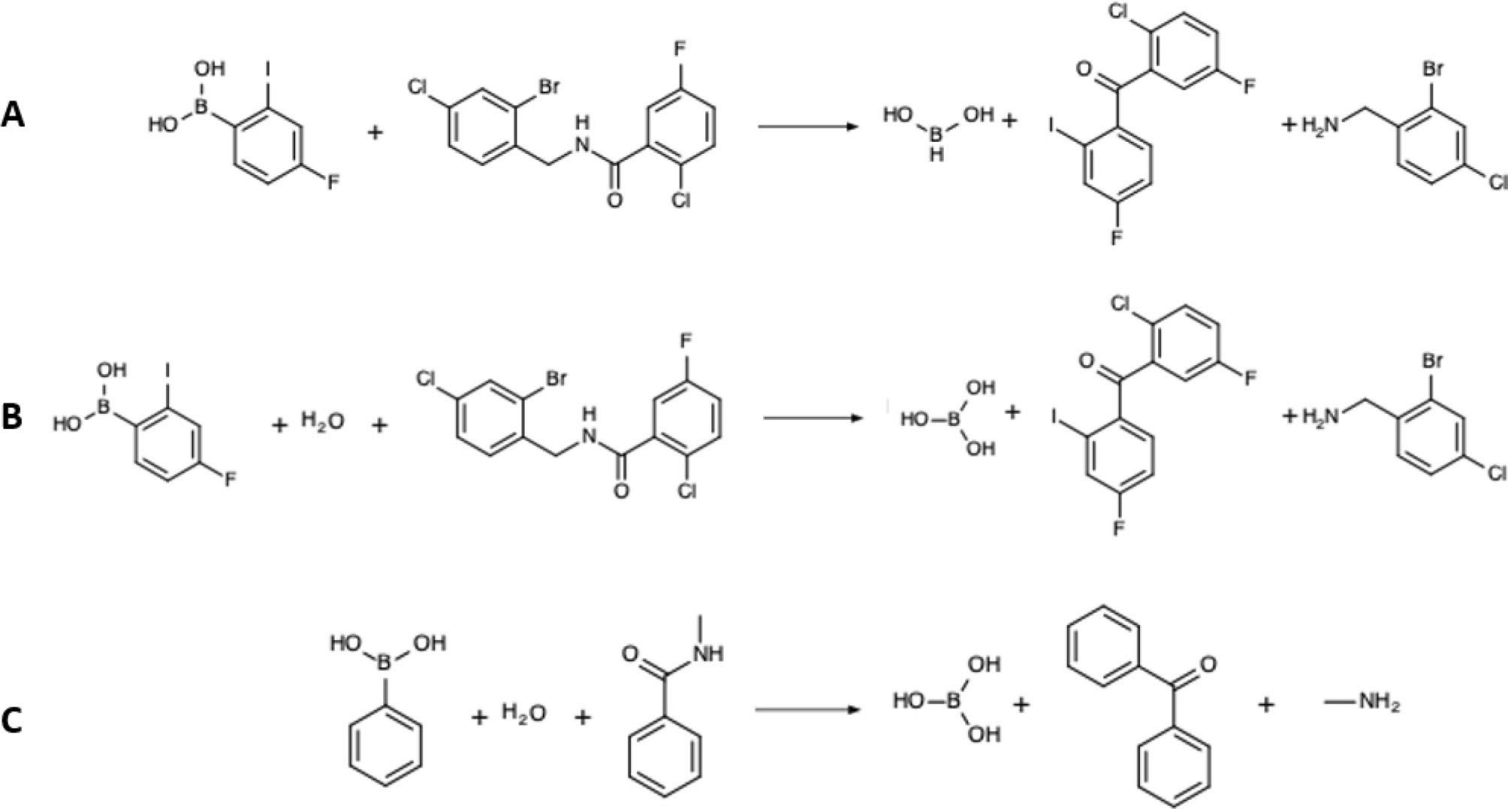
Example of generated chemical reaction with a new reaction center as is (**A**), balanced by the addition of a water molecule as a reactant (**B**), and its simplified form (**C**). Notice that the aminobenzylic leaving group suggested by the autoencoder for generated reaction looks unrealistic.

Output of unbalanced reactions is a direct consequence of the training set composition: almost all USPTO reactions are also unbalanced, e.g., leaving groups are almost never reflected in the reaction equation present in the database. The application of the CGR technology may implicitly solve that problem. Indeed, within the CGR formalism,heavy atoms in reactants and products are implicitly conserved—as the same graph is simply interpreted differently in terms of dynamical bond status in order to convert it to reagents or to products, respectively. Even if the initial reaction was not stoichiometrically balanced (see example in SI), its CGR representation will be—in so far the conversion of an unbalanced transformation to CGR succeeds to produce the correct CGR of the balanced process. However, as the exact state of the leaving group cannot be deduced from the training set, in silico generated CGRs may occasionally decode into reactions by simply substituting a broken bond by a hydrogen atom, leading to a disbalance in terms of implicit hydrogens.This is seen in the example from Fig. 3A in which the products contain 2 hydrogens more than reactants. Furthermore, the postulated product BH(OH)_2_ is highly reactive, thus unrealistic. Formally, this is a rather “creative” in silico interpretation of the Suzuki reaction pattern, in which the organic halide R–X is replaced by an amide group: the acyl fragment is assimilated to “R” while the benzylamine is the leaving group X. Formally, a balanced Suzuki process could be formulated as either

1 $$R - X \, + \, R^{\prime} - B{\left( {OH} \right)_2} \to R - R^{\prime} + \, X - B{\left( {OH} \right)_2}$$

or, more realistically, with inclusion of the required alkoxy base, typically BuO^-^:

2 $$R - X \, + \, R^{\prime} - B{\left( {OH} \right)_2} + \, Bu{O^- } \to R - R^{\prime} + \, BuO - B{\left( {OH} \right)_2} + \, {X^- }$$

Unfortunately, a sketchily written Suzuki transformation, carelessly ignoring the inorganic leaving groups:

3 $$R - X \, + \, R^{\prime} - B{\left( {OH} \right)_2}\; \to R - R^{\prime}$$

converts to a CGR corresponding to

4 $$R - X \, + \, R^{\prime} - B{\left( {OH} \right)_2} \to R - R^{\prime} + \, X \, + \, B{\left( {OH} \right)_2}$$

in which the unsatisfied valences of X and B are interpreted by chemoinformatics tools as implicit hydrogens. This explains why the AI tool is inclined to generate formal reactions of type (4), which are nothing but a biased interpretation of Suzuki processes, corrupted by intrisic representation errors in USPTO database entries. The addition of a water molecule as a “formal” basic species leads to a fully balanced reaction (Fig. 3B). Although water is not a perfect base for the Suzuki reaction, it helps to correctly represent the boron-containing leaving group in reaction equations. In silico generated reactions that cannot be “corrected” in this way have been discarded.

We also decided to discard the unfeasible under normal conditions transformations consisting in the cleavage of a C–C bond and assuming a carbon-centric leaving group. Application of these heuristics reduced a considered set of novel reactions to 44 including 31 reactions with new ***RC*** and 13 reactions with new ***RC + 1***.

The question arises whether we need to consider explicit chemical structures of generated reactants and products. In our opinion, this is not firmly required if one focuses on the detection of new reaction transformations identified by ***RC*** or ***RC + 1*** structural motifs. In this case, a “simplified” reaction in which substrates contain only atoms of reaction center and their closest environment (including second neighbors) could be sufficient, see Fig. 3C. Notice that such simplified reactions correspond to general reactivity patterns. Particular reactants can be selected by chemists as a function of availability, intended conditions, reactivity concerns, etc. For example, the reaction in Fig. 3C looks unfeasible, but it becomes more realistic if the amine leaving group were strengthened by binding to strong electron acceptors (for example, trifluoromethanesulfonyl) or by quaternization.

Notice that the majority of generated reactions have known ***RC*** and ***RC + 1*** motifs. All belong to the Suzuki coupling type, as exemplified below.
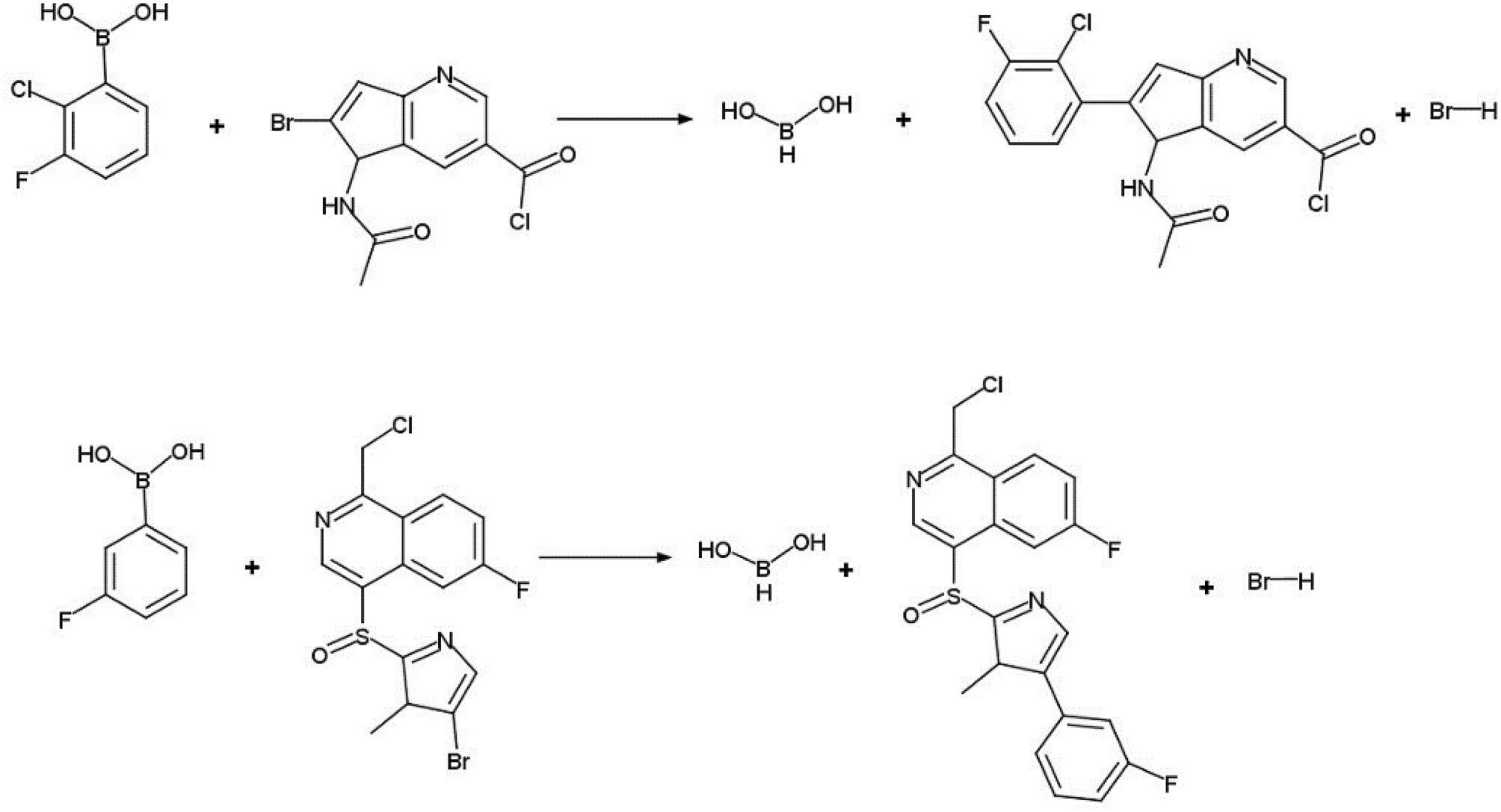


### Reactions with new reaction centers

31 reactions featuring a total of 13 distinct reaction centers not seen in USPTO were generated (see Table 1 and Table S3 in Supporting Information). Substructural searching with ***RC*** as a query in the much larger CAS REACT database (SciFinder) resulted in retrieval of several reactions similar to those “discovered” by Artificial Intelligence. In particular, this concerns reactions with C–Br^56^ bond formation and C–Si^57^ coupling, and C–C coupling with N-containing^58^ and F^59^ leaving groups, as well as C–O coupling with organosilicon leaving groups. In total, 5 out of 13 new reaction centers discovered computationally, were found in SciFinder reactions (Table S5 in Supporting Information). Since none of them were used for the autoencoder training, these generated reactions were pure “imagination” of AI. Thus, several “novel” reactions (4 in Table 1, 5, and 7 in Table S5) correspond to a quite interesting C-N bond cleavage with amine as leaving group. A similar reaction has recently been discovered experimentally by Weires et al.^60^ who shown that the formation of amides facilitated nickel-catalyzed cleavage of C–N bonds accompanied by C–C coupling (reaction 12 in Table S3). Experimental analogues of C-Si coupling reactions generated by the model (reactions 8 and 9 in Table 1, reactions 19–26 in Table S3) were found in SciFiner (reaction 9 in Table 1 and 19 in Table S5). In the experiment, bromotriarylsilane was used as template^57^ (reaction 19 in Table S5) whereas our tool proposed less stable di-substituted silane bromide possibly with heteroatoms surrounding silicon (reactions 8 and 9 in Table 1). The organosilicon leaving group proposed for the C-O coupling (reaction 11 in Table 1) is very similar to that reported by Kori et al.^61^ Fluoro-Suzuki reaction proposed by the autoencoder (reaction 5 in Table 1) was observed experimentally in the study by Chi et al.^62^ Reaction 7 in Table 1 is not a coupling but boron substitution by bromine; it has been experimentally discovered using N-bromosuccinimide as a donor of bromine in the study by Thiebes et al^56^ (reaction 17 in Table S5). However, from the structural point of view, its reaction center looks similar to “classical” Suzuki type reactions (boron substitution by carbon or heteroatom).

**Table 1:** Examples of simplified Suzuki-type reactions with a new reaction center together with corresponding conventional reaction SMILES notation.

	Reaction center SMILES	Simplified reaction	References
1	O.BC.N > > OB.CN		*a*
2	O.BC.OI > > OB.CO.I		*a*
3	O.BC.CS > > OB.CC.S		*a*
4	O.BC.CN > > OB.CC		^58^
5	O.BC.CF > > OB.CC.F		^59^
6	O.BC.[Si]S > > OB.C[Si].S		*a*
7	O.BC.BrN > > BO.CBr.N		^56^
8	O.BC.[Si]Br > > OB.C[Si].Br		*a*
9	O.BC.[Si]Br > > OB.C[Si].Br		^57^
10	O.BC.SBr > > OB.CS.Br		*a*
11	O.BC.O[Si] > > OB.CO.[Si]		^61^

The right column refers to similar types of reactions found in SciFinder. A complete list of simplified reactions is given in SI.

^a^Reaction centers for which no information in the literature was found.

Some of the reactions still look unfeasible, e.g., the O–I compound seems quite unstable (reaction 2 in Table 1). Nonetheless, such compounds are listed as commercially available (e.g. CAS Nos 3240-34-4, 1338247-47-4). Sulfur-containing compounds are generally unsuitable for Suzuki catalysts. Their generation can be explained by an excessive model’s “creativity”, which can be hardly controlled in the employed neural network architecture.

### Reactions with a new environment of known reaction centers (RC + 1)

Following the novelty detection procedure, 13 reactions that correspond to 3 known reaction centers but an original first environment (***RC + 1***) were detected, see Table 2 and Table S4 in SI. Two similar reactions have been found in SciFinder. Although the simplified reaction 1 in Table 2 looks unfeasible, a more suitable leaving group might render it possible. For instance, in hydrogenation conditions, a catalyst can facilitate reductive cleavage of C–O bond (in esters, carbamates, benzyl ethers, etc.) followed by a coupling (as in reaction 10 in Table S6).

**Table 2:** Examples of simplified Suzuki-type reactions with the **RC** present in the training set but in a new chemical environment.

	Reaction center SMILES	Simplified reaction	References
1	BC.O.CO > > O.CC.BO		^64^
2	BC.O.CI > > I.CC.BO		^63^
3	BC.O.CBr > > Br.CC.BO		^65^

The right column refers to similar types of reactions found in SciFinder. A complete list of simplified reactions is given in SI.

The use of alkyl and acyl bromides in C–C coupling in reaction 3 (Table 2), was observed experimentally (see reaction 13 in Table S6 in SI). Reaction 2 in Table 2 looks quite feasible because synthesis of acyl iodides was reported in the literature (e.g., CAS 191340-22-4 and CAS 1332596-80-1) whereas carboniodidates can be provided by some vendors (e.g., Enamine BBV-109267542 or BBV-109267541). Notice that similar reactions with chloroformates have been also found in Reaxys^63^.

### Reactions feasibility assessment

Strictly speaking, reaction feasibility is defined by both kinetic and thermodynamic factors. However, according to the Bell-Evans-Polanyi principle^66,67^, in a series of similar reactions, the trend of activation energies follows the trend of reaction enthalpies. Thus, favorable thermodynamics, namely reaction enthalpy (∆H), can be considered as weak proof of reaction feasibility. A series of gas-phase DFT calculations were performed to assess ∆H for all simplified reactions with new ***RC*** and ***RC + 1***. According to our estimations, almost all reactions are exothermic except for four reactions with Si-containing substrates in which ∆H is positive but close to zero (see Tables S3 and S4 in Supporting Information). This shows that all new computer-generated reactions are feasible, at least, as far as DFT-based thermodynamics estimates can tell. Since DFT is a rather time-consuming method and can hardly be applied for thousands of generated reactions, we also performed a rough estimation of ∆H using the tabulated bond energies in reactants and products^68–70^. Although calculated in such a way reaction enthalpies poorly correlate with the DFT values, they generally provide similar conclusions concerning reaction feasibility (see Tables S3 and S4 in Supporting Information).

## Conclusion

Here we present the first attempt to generate new chemical reactions using a combination of Condensed Graph of Reaction, Generative Topographic Mapping, and sequence-to-sequence autoencoder. To feed the autoencoder, special reaction SMILES strings (SMILES/CGR) were conceived and implemented. In order to discard the seemingly unfeasible reactions, a special 4-steps post-processing procedure has been implemented. It includes: (i) stoichiometric balancing of reaction equations, (ii) reduction of substrates structure to their simplified form, (iii) discarding chemically infeasible transformations using suggested heuristics (“Chemical filters”), and (iv) assessment of synthetic feasibility using quantum mechanics calculations. The effectivness of the suggested approach was demonstrated on the example of Suzuki-like coupling reactions. Among generated reactions we discovered transformations with 13 new reaction centers which did not occur in the training set. Five out of 13 transformations were then found in the reaction databases (not used in the model training), thus showing the reliability of our approach to generate new synthetically feasible reactions.

This study reveals that creativity of Artificial Intelligence is rather limited. Deep learning neural networks, at least, in their current state, are not able to invent completely new type of chemical transformations but rather propose unseen and sometimes not trivial variations of existing ones. Thus, in this study novel (in the context of the training set) C–N, C–O, C–S and C–Si bond formation reactions, as well as nitrogen- and sulfur-containing leaving groups have been suggested by the model. We believe that this opens a way to propose putatively new synthetic pathways in a way that is not affected by the bias of human expertise—with all the benefits and the pitfalls this may bring. It should also be noted that compared to theory-driven quantum chemical models, data-driven DNN is much less time consuming and, practically, is not limited by the reactants size. The more data are used in the neural network training, the more realistic the predicted reactions are. Since the sizes of reaction databases are rapidly growing up, deep learning approach has an obvious perspective as a tool for discovery of novel reactions.

## Methods

### Datasets and data curation

The dataset used in this project comes from United States Patents and Trademark Office database (1976 to 2016) extracted by Lowe^53^. It contains about 3.5 million reactions. The initial dataset was preprocessed with *in-house* scripts based on the CGRtools library^51^. The curation includes the standardization (aromatization and functional group standardization), removal of empty reactions (those where the products and reactants are the same, or no reactants or products are recorded) and reactions with valence errors. For curated reactions, atom-to-atom mapping (AAM) was performed using the ChemAxon Automapper tool which is a part of the JChem toolkit^71^. The mapped reactions were converted into CGRs and their reaction centers were extracted with the CGRtools. In total, 165 879 different reaction centers were obtained. Since AAM errors lead to incorrect reaction centers, which are usually rare, only highly populated reaction centers were selected. Thus, the resulting dataset consisted of some 2.5 million reactions (approximately 70% of the initial dataset) which corresponds to 300 most frequent reaction centers.

According to our estimations^72^, the ChemAxon Automapper tool leads to the erroneous AAM for some 25% of USPTO reactions. Most of those concern cycloadditions with complex reaction centers. As far as Suzuki coupling is concerned, this error is around 3%.

Notice that practically all USPTO reactions are stoichiometrically unbalanced. This doesn’t prevent to build Condensed Graph of Reaction, but, in some cases, may lead to erroneous atom-to-atom mapping.

### Reaction data treatment

CGRtools library (version 3)^51^ was used for the reactions cleaning, their transformation to CGRs, conversion of CGRs into SMILES/CGR, and processing of generated SMILES/CGR back into reactions.

### SMILES/CGR notation

Generally, SMILES/CGR follows the OpenSMILES rules^73^. They differ from regular Daylight SMILES in terms of aromatic atoms and ring closure specification and introduce special “dynamic” bonds and atoms characterizing chemical transformations. Dynamic bonds in CGR characterizing chemical transformations have special labels representing changes in bond orders. Dynamic atom corresponds to change of formal charge or radical state of this atom in reaction. Detailed information about SMILES/CGR syntax is given in Supporting Information. SMILES/CGR generation and parsing, including preparation of canonic SMILES/CGR, are implemented into CGRtools Python library^51^.

### Reaction generation algorithm

The network architecture previously applied for molecular SMILES generation^30^ has been used in this study. It is based on the autoencoder architecture introduced by Xu et al.^74^. SMILES/CGR transformed into sequences of one-hot encoded characters with padding to constant length (256) were used to feed the encoder. Symbols within square brackets (conventional or dynamic atoms or dynamic bonds) were considered as a single symbol within tokenization. The encoder consists of two bidirectional Long Short-Term Memory (LSTM)^52^ layers (128 nodes each), while the decoder is composed of two forward LSTM layers (256 nodes each). The bottleneck dense layer between the encoder and the decoder transforms the states of the encoder LSTMs into latent variables to subsequently feed them to the decoder; it consists of 128 nodes. Finally, the decoder outputs are transformed back to one-hot encoded characters via a single dense layer.

The autoencoder was trained in batch mode, where batches of “one-hot”-encoded sequences were generated on-the-fly from training set SMILES/ CGR strings. The Adam optimizer was used for training, initial learning rate was set to 0.005, and batch size was set to 256 samples per batch. The learning rate was reduced during training if there were no improvement in the validation loss for two epochs. The training was terminated after 34 epochs when no improvements in test set reconstruction accuracy was observed. To generate latent variable vectors for eventual decoding, we use the Generative Topographic Mapping method. It is a non-linear dimensionality reduction method that has been successfully used for chemical space analysis^54,74–82^, comparison of chemical libraries^83^, building classification^43,74–77,80,84^, and regression^85,86^ models via activity landscapes, as well as for solving the “inverse” QSAR problem^87^. The GTM algorithm operates by embedding a nonlinear two-dimensional manifold into a D-dimensional descriptor space and calculating the distribution of objects of initial space on these two dimensions. In this work, we utilize the autoencoder’s latent vectors as an initial descriptor space. Once a map for the entire USPTO database was constructed, the zones corresponding to the desired reaction type (Suzuki reaction) were located, from which the latent vectors for virtual reactions were sampled. These new vectors fed the trained decoder resulting in new SMILES/CGR strings.

### Novelty detection

**Figure 4:**
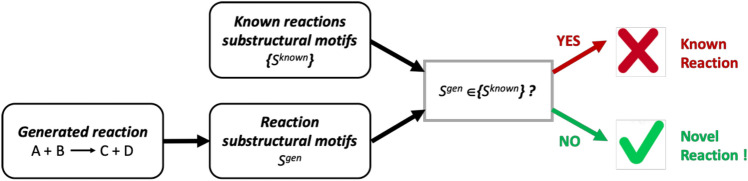
Reactions novelty detection workflow. Substructural motifs *S*^*gen*^ (***RC****, ****RC + 1****, ****RC + 2,*** …) are extracted from the query CGR and compared with those for known reactions {*S*^*known*^}. In such a way, motifs belonging to novel reactions will easily be identified.

**Figure 5:**
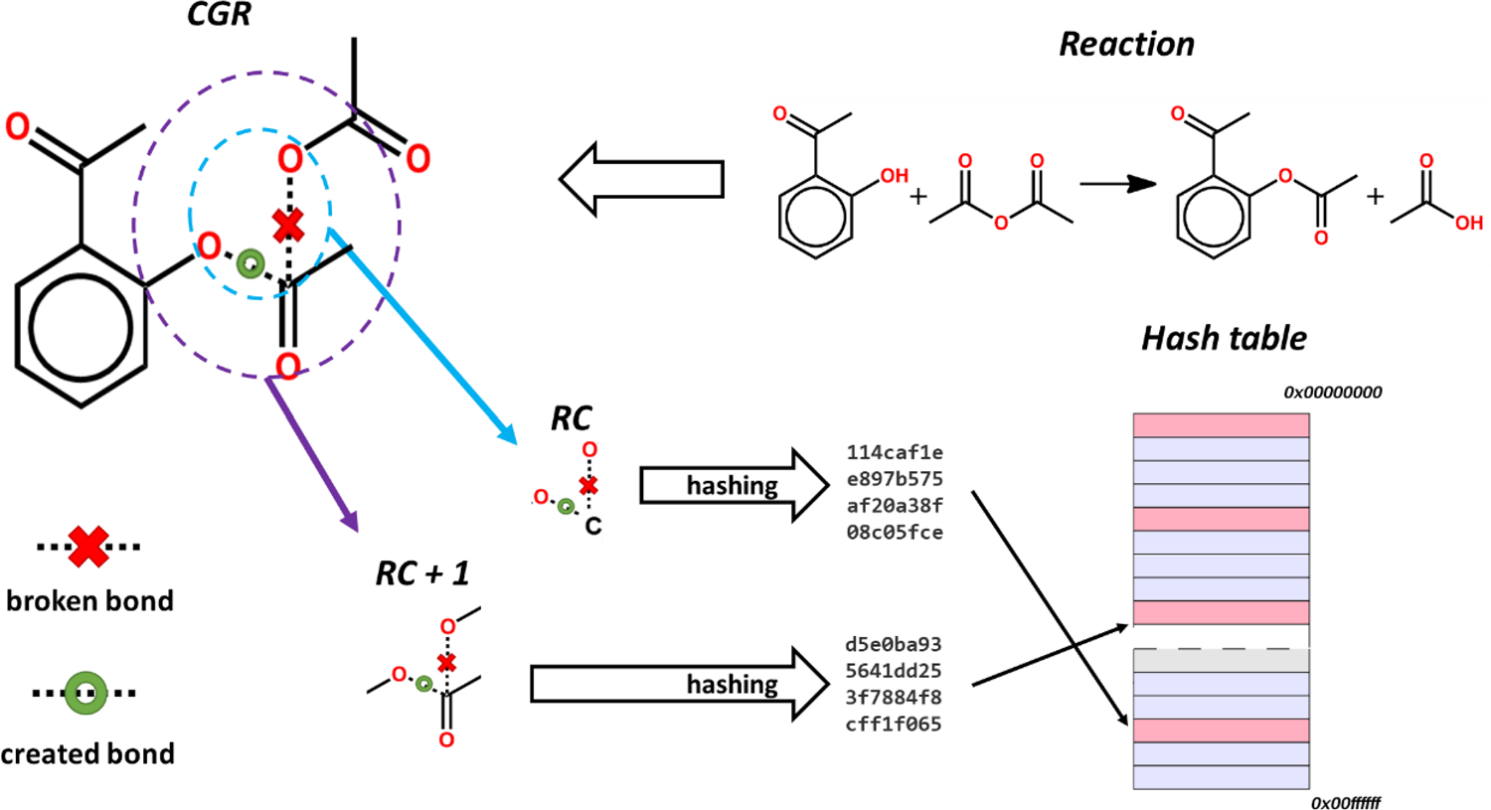
Preparation of a collection of reaction signatures as hash codes. From a CGR generated from a given reaction, substructural motifs containing reaction center (RC), or reaction center with *n* neighboring bonds and atoms (RC + *n*, here *n* = 1) can be extracted. Each motif is encoded by a hashing function into a unique hash code—reaction signature. The ensemble of unique hash codes for all reactions in the database is stored in the hash table.

Novelty detection is based on the comparison of hashed reaction signatures corresponding to reaction centers (**RC**) and their environment between the database of known reaction (here, USPTO database) and the reactions generated by the autoencoder (Fig. 4). Encoding chemical reactions by CGR significantly simplifies ***RC*** detection. Thus, substructural motifs involving the reaction center (***RC****, ****RC + 1****, ****RC + 2,*** …) can easily be extracted from CGR (see Fig. 5). Since any operations with molecular graphs are time-consuming, each substructural motif was encoded by a unique hash code^51^—a reaction signature uniquely identifying given transformation. In this case, the novelty detection is reduced to the comparison of signature (hash code) of a generated reaction with those of known reactions (Fig. 4). The suggested procedure assures fast and precise novelty detection.

### Reaction enthalpy calculations

The difference in energies between reactants and products is calculated in several steps^88^. First, a conformer with the lowest energy is generated for each compound in the reaction using the ChemAxon cxcalc module. Then, the geometry of each compound was optimized using the Priroda16 program with PBE exchange and correlation functional^89^, and the built-in triple-zeta split valence basis set 3z, which is equivalent to Schäfer’s TZVP basis set^90^. Relativistic and solvent effects were neglected. The Priroda16 program was chosen as it is one of the fastest DFT software due to the efficient evaluation of density functional exchange–correlation terms based on the electron density expansion^91^. Final energy values were extracted for optimized structures and used for calculation of reaction enthalpy. The additive scheme for estimating reaction enthalpies was implemented using the tabulated chemical bonds increments^68–70^.

## Supplementary Information

Supplementary Information

## Data Availability

The dataset used in this project comes from the publicly available United States Patents and Trademark Office database (Lowe, https://doi.org/10.17863/CAM.16293). Curated USPTO dataset is available on GitHub: https://github.com/Laboratoire-de-Chemoinformatique. All data preprocessing procedures are described in the Methods section and are based on freely available CGRtools library.

## Code availability

CGRtools library is used for data preprocessing and creation and treatment of chemical reactions as SMILES/CGR, and is freely available (https://github.com/cimm-kzn/CGRtools). Autoencoder model code and the ISIDA/GTM tool are available upon request.

## References

[1] Herges R (1988). Reaction planning: Computer-aided reaction design. Tetrahedron Comput. Methodol..

[2] Balaban AT (1967). Chemical graphs. 3. Reactions with cyclic 6-membered transition states. Rev. Roum. Chim..

[3] Hendrickson JB (1974). The variety of thermal pericyclic reactions. Angew. Chem. Int. Ed. English.

[4] Arens JF (1979). A formalism for the classification and design of organic reactions. I. The class of (− +)n reactions. Recl. des Trav. Chim. des Pays-Bas.

[5] Arens JF (1979). A formalism for the classification and design of organic reactions. II. The classes of (+ −)n + and (− +)n − reactions. Recl. des Trav. Chim. des Pays-Bas.

[6] Arens JF (1979). A formalism for the classification and design of organic reactions III. The class of (+ - )nC reactions. Recl. des Trav. Chim. des Pays-Bas.

[7] Zefirov NS, Tratch SS (1977). Formal-logical approach to multicentered processes with cyclic electron transfer. Match.

[8] Zefirov NSS, Tratch SSS, Trach SS (1980). Systematization of tautomeric processes and formal-logical approach to the search for new topological and reaction types of tautomerism. Chem. Scr..

[9] Bauer J, Herges R, Fontain E, Ugi I (1985). IGOR and computer assisted innovation in chemistry. Chimia (Aarau)..

[10] Bauer J (1989). IGOR2: A PC-program for generating new reactions and molecular structures. Tetrahedron Comput. Methodol..

[11] Dugundji J, Ugi I (1973). An algebraic model of constitutional chemistry as a basis for chemical computer programs. Computers in Chemistry.

[12] Herges R (1990). Reaction planning: Prediction of new organic reactions. J. Chem. Inf. Comput. Sci..

[13] Herges R, Hoock C (2020). Reaction planning: Computer-aided discovery of a novel elimination reaction. Science.

[14] Zefirov NS, Baskin II, Palyulin VA (1994). SYMBEQ program and its application in computer-assisted reaction design. J. Chem. Inf. Comput. Sci..

[15] Zefirov N, Tratch S, Molchanova M (2002). The argent program system: A second-generation tool aimed at combinatorial search for new types of organic reactions. Math. Comput. Chem..

[16] Molchanova MS, Tratch SS, Zefirov NS (2003). Computer-aided design of new organic transformations: Exposition of the ARGENT-1 program. J. Phys. Org. Chem..

[17] Baskin II, Madzhidov TI, Antipin IS, Varnek AA (2017). Artificial intelligence in synthetic chemistry: Achievements and prospects. Russ. Chem. Rev..

[18] Segler S, Marwin HS, Preuss M, Waller MP (2018). Planning chemical syntheses with deep neural networks and symbolic AI. Nature.

[19] Sutskever I, Vinyals O, Le QV (2014). Sequence to sequence learning with neural networks. Adv. Neural. Inf. Process. Syst..

[20] Nam, J. & Kim, J. Linking the Neural Machine Translation and the Prediction of Organic Chemistry Reactions. Preprint at *arXiv* http://arxiv.org/abs/1612.09529 (2016).

[21] Schwaller P, Gaudin T, Lányi D, Bekas C, Laino T (2018). “Found in Translation”: Predicting outcomes of complex organic chemistry reactions using neural sequence-to-sequence models. Chem. Sci..

[22] Liu B (2020). Retrosynthetic reaction prediction using neural sequence-to-sequence models. ACS Cent. Sci..

[23] Karpov P, Godin G, Tetko IV (2019). A transformer model for retrosynthesis. Lect. Notes Comput. Sci..

[24] Schwaller, P. *et al.* Predicting retrosynthetic pathways using a combined linguistic model and hyper-graph exploration strategy. (2019) doi:10.26434/chemrxiv.9992489.v1. 10.1039/c9sc05704h PMC8152799 34122839

[25] Coley CW, Barzilay R, Jaakkola TS, Green WH, Jensen KF (2017). Prediction of organic reaction outcomes using machine learning. ACS Cent. Sci..

[26] Fooshee D (2018). Deep learning for chemical reaction prediction. Mol. Syst. Des. Eng..

[27] Kayala MA, Baldi P (2012). ReactionPredictor: Prediction of complex chemical reactions at the mechanistic level using machine learning. J. Chem. Inf. Model..

[28] Xue D (2019). Advances and challenges in deep generative models for de novo molecule generation. Wiley Interdiscip. Rev. Comput. Mol. Sci..

[29] Xu Y (2019). Deep learning for molecular generation. Fut. Med. Chem..

[30] Sattarov B (2019). De novo molecular design by combining deep autoencoder recurrent neural networks with generative topographic mapping. J. Chem. Inf. Model..

[31] Elton DC, Boukouvalas Z, Fuge MD, Chung PW (2019). Deep learning for molecular design—a review of the state of the art. Mol. Syst. Des. Eng..

[32] Blaschke T, Olivecrona M, Engkvist O, Bajorath J, Chen H (2018). Application of generative autoencoder in de novo molecular design. Mol. Inform..

[33] Sanchez-Lengeling B, Aspuru-Guzik A (2018). Inverse molecular design using machine learning: Generative models for matter engineering. Science (80-).

[34] Jørgensen PB, Schmidt MN, Winther O (2018). Deep generative models for molecular science. Mol. Inform..

[35] Gupta A (2018). Generative recurrent networks for de novo drug design. Mol. Inform..

[36] Segler MHS, Waller MP (2017). Modelling chemical reasoning to predict and invent reactions. Chem. A Eur. J..

[37] Brown N, Fiscato M, Segler MHS, Vaucher AC (2019). GuacaMol: Benchmarking models for de novo molecular design. J. Chem. Inf. Model..

[38] Hoonakker F, Lachiche N, Varnek A, Wagner A (2011). A representation to apply usual data mining techniques to chemical reactions illustration on the rate constant of SN2 reactions in water. Int. J. Artif. Intell. Tools.

[39] Varnek A, Fourches D, Hoonakker F, Solovev VP (2005). Substructural fragments: An universal language to encode reactions, molecular and supramolecular structures. J. Comput. Aided. Mol. Des..

[40] Hoonakker F, Lachiche N, Varnek A, Wagner A (2010). A representation to apply usual data mining techniques to chemical reactions. Lect. Notes Comput. Sci..

[41] Madzhidov TI (2014). Structure-reactivity relationships in terms of the condensed graphs of reactions. Russ. J. Org. Chem..

[42] Madzhidov TII (2015). Structure-reactivity relationship in bimolecular elimination reactions based on the condensed graph of a reaction. J. Struct. Chem..

[43] Gimadiev T (2019). Bimolecular nucleophilic substitution reactions: Predictive models for rate constants and molecular reaction pairs analysis. Mol. Inform..

[44] Glavatskikh M (2019). Predictive models for kinetic parameters of cycloaddition reactions. Mol. Inform..

[45] Gimadiev TRR (2018). Assessment of tautomer distribution using the condensed reaction graph approach. J. Comput. Aided. Mol. Des..

[46] Gimadiev, T. R. *et al.* Prediction of tautomer equilibrium constants using condensed graphs of reaction. in *Second Kazan Summer School on Chemoinformatics* 34 (2015).

[47] Horvath D (2016). Prediction of activity cliffs using condensed graphs of reaction representations, descriptor recombination, support vector machine classification, and support vector regression. J. Chem. Inf. Model..

[48] Latino DARS, Aires-de-Sousa J (2011). Classification of chemical reactions and chemoinformatic processing of enzymatic transformations. Methods Mol. Biol..

[49] Madzhidov, T. I. *et al.* Artificial neural networks model for assessment of optimal conditions of hydrogenation reactions. in *In 22nd European Symposium on Quantitative Structure-Activity Relationships.* 186 (2018).

[50] Marcou G (2015). Expert system for predicting reaction conditions: The michael reaction case. J. Chem. Inf. Model..

[51] Nugmanov RI (2019). CGRtools: Python library for molecule, reaction, and condensed graph of reaction processing. J. Chem. Inf. Model..

[52] Hochreiter S, Schmidhuber J (1997). Long short-term memory. Neural Comput..

[53] Lowe, D. M. M. Extraction of chemical structures and reactions from the literature. *Doctoral Thesis* (University of Cambridge, 2012). doi:https://doi.org/10.17863/CAM.16293.

[54] Gaspar HA (2016). Generative topographic mapping approach to chemical space analysis. ACS Symp. Ser..

[55] Chen WL, Chen DZ, Taylor KT (2013). Automatic reaction mapping and reaction center detection. Wiley Interdiscip. Rev. Comput. Mol. Sci..

[56] Thiebes C, Thiebes C, Prakash GKS, Petasis NA, Olah GA (1998). Mild preparation of haloarenes by ipso-substitution of arylboronic acids with N -halosuccinimides. Synlett.

[57] Park, J. *et al.* Indole compound, compound for organic electric element containing derivative thereof, organic electric element using same, and corresponding electronic device. PCT/KR2013/003289. (2013).

[58] Zong Y, Hu J, Sun P, Jiang X (2012). Synthesis of biaryl derivatives via a magnetic Pd-NPs-catalyzed one-pot diazotization–cross-coupling reaction. Synlett.

[59] Luo Z-J, Zhao H-Y, Zhang X (2018). Highly selective Pd-catalyzed direct C–F bond arylation of polyfluoroarenes. Org. Lett..

[60] Weires NA, Baker EL, Garg NK (2016). Nickel-catalysed Suzuki-Miyaura coupling of amides. Nat. Chem..

[61] Kori M (2012). Fused thiadiazine derivatives as AMPA receptor potentiators and their preparation and use for the treatment of diseases. PCT Int. Appl..

[62] Chi, Y. & Lin, J. Iridium complex, OLED using the same, and nitrogen-containing tridentate ligand having carbene unit. *Faming Zhuanli Shenqing* 106928281 https://patents.google.com/patent/US10153442B2 (2017).

[63] Duan Y-Z, Deng M-Z (2005). Palladium-catalyzed cross-coupling reaction of arylboronic acids with chloroformate or carbamoyl chloride. Synlett.

[64] Dindarloo Inaloo, I., Majnooni, S., Eslahi, H. & Esmaeilpour, M. Nickel(II) Nanoparticles Immobilized on EDTA-Modified Fe3O4.SiO2 Nanospheres as Efficient and Recyclable Catalysts for Ligand-Free Suzuki–Miyaura Coupling of Aryl Carbamates and Sulfamates. *ACS Omega* **5**, 7406–7417 (2020). 10.1021/acsomega.9b04450 PMC7144170 32280882

[65] Chakraborty J, Nath I, Verpoort F (2019). Pd-nanoparticle decorated azobenzene based colloidal porous organic polymer for visible and natural sunlight induced Mott-Schottky junction mediated instantaneous Suzuki coupling. Chem. Eng. J..

[66] Bell RP, Hinshelwood CN (1936). The theory of reactions involving proton transfers. Proc. R. Soc. London. Ser. A Math. Phys. Sci..

[67] Evans MG, Polanyi M (1936). Further considerations on the thermodynamics of chemical equilibria and reaction rates. Trans. Faraday Soc..

[68] Cottrell, T. L. *The strengths of chemical bonds*. (Butterworths Scientific Publications, 1958).

[69] Darwent, B. deB. *Bond dissociation energies in simple molecules*. (1970).

[70] Benson SW (1965). Bond energies. J. Chem. Educ..

[71] ChemAxon. Chemical Structure Representation Toolkit. (2019).

[72] Lin, A. I. *et al.* Atom-to-Atom Mapping: A Benchmarking Study of Popular Mapping Algorithms and Consensus Strategies. 10.26434/chemrxiv.13012679.v1 (2020). 10.1002/minf.202100138 34726834

[73] James, C. A. OpenSMILES specification. www.opensmiles.org (2016).

[74] Xu, Z., Wang, S., Zhu, F. & Huang, J. Seq2seq Fingerprint. in *Proceedings of the 8th ACM International Conference on Bioinformatics, Computational Biology,and Health Informatics - ACM-BCB ’17* 285–294 (ACM Press, 2017). doi:10.1145/3107411.3107424.

[75] Gimadiev TR, Madzhidov TI, Marcou G, Varnek A (2016). Generative topographic mapping approach to modeling and chemical space visualization of human intestinal transporters. Bionanoscience.

[76] Klimenko K, Marcou G, Horvath D, Varnek A (2016). Chemical space mapping and structure-activity analysis of the ChEMBL antiviral compound set. J. Chem. Inf. Model..

[77] Sidorov P, Gaspar H, Marcou G, Varnek A, Horvath D (2015). Mappability of drug-like space: Towards a polypharmacologically competent map of drug-relevant compounds. J. Comput. Aided. Mol. Des..

[78] Maniyar DM, Nabney IT, Williams BS, Sewing A (2006). Data visualization during the early stages of drug discovery. J. Chem. Inf. Model..

[79] Owen JR, Nabney IT, Medina-Franco JL, López-Vallejo F (2011). Visualization of molecular fingerprints. J. Chem. Inf. Model..

[80] Kireeva N (2012). Generative topographic mapping (GTM): Universal tool for data visualization, structure-activity modeling and dataset comparison. Mol. Inform..

[81] Glavatskikh M (2018). Visualization and analysis of complex reaction data: The case of tautomeric equilibria. Mol. Inform..

[82] Horvath, D., Marcou, G. & Varnek, A. Generative topographic mapping approach to chemical space analysis. 167–199 (2017). doi:10.1007/978-3-319-56850-8_6.

[83] Gaspar HA, Baskin II, Marcou G, Horvath D, Varnek A (2015). Chemical data visualization and analysis with incremental generative topographic mapping: Big data challenge. J. Chem. Inf. Model..

[84] Gaspar HA (2013). Generative topographic mapping-based classification models and their applicability domain: Application to the biopharmaceutics drug disposition classification system (BDDCS). J. Chem. Inf. Model..

[85] Gaspar HA, Baskin II, Marcou G, Horvath D, Varnek A (2015). GTM-based QSAR models and their applicability domains. Mol. Inform..

[86] Baskin AA, Solovev VP, Bagaturyants AA, Varnek A (2017). Predictive cartography of metal binders using generative topographic mapping. J. Comput. Aided. Mol. Des..

[87] Gaspar HA, Baskin II, Marcou G, Horvath D, Varnek A (2015). Stargate GTM: Bridging descriptor and activity spaces. J. Chem. Inf. Model..

[88] Gimadiev TR, Klimchuk O, Nugmanov RI, Madzhidov TI, Varnek A (2019). Sydnone-alkyne cycloaddition: Which factors are responsible for reaction rate ?. J. Mol. Struct..

[89] Perdew JP, Burke K, Ernzerhof M (1996). Generalized gradient approximation made simple. Phys. Rev. Lett..

[90] Schäfer A, Huber C, Ahlrichs R (1994). Fully optimized contracted Gaussian basis sets of triple zeta valence quality for atoms Li to Kr. J. Chem. Phys..

[91] Laikov DN (1997). Fast evaluation of density functional exchange-correlation terms using the expansion of the electron density in auxiliary basis sets. Chem. Phys. Lett..

