# Supplementary material for: Discovery of novel chemical reactions by deep generative recurrent neural network
Source: Sci Rep. 2021 Feb 4;11:3178. doi: 10.1038/s41598-021-81889-y (PMC7862614; doi:10.1038/s41598-021-81889-y)
Supplement: Supplementary file 1 — Supplementary Information [file 41598_2021_81889_MOESM1_ESM.docx]

# Discovery of Novel Chemical Reactions by Deep Generative Recurrent Neural Network

William Bort^1^, Igor I. Baskin^1,3^, Timur Gimadiev^4^, Artem Mukanov^2^, Ramil Nugmanov^2^, Pavel Sidorov^4^, Gilles Marcou^1^, Dragos Horvath^1^, Olga Klimchuk^1^, Timur Madzhidov^2^, and Alexandre Varnek^1,4^ *

^1^ Laboratory of Chemoinformatics, UMR 7140 CNRS, University of Strasbourg, 1, rue Blaise Pascal, 67000 Strasbourg, France

^2^ Laboratory of Chemoinformatics and Molecular Modeling, Butlerov Institute of Chemistry, Kazan Federal University, Kremlyovskaya str. 18, 420008 Kazan, Russia

^3^ Faculty of Physics, M.V. Lomonosov Moscow State University, Leninskie Gory, 119991 Moscow, Russia

^4^ Institute for Chemical Reaction Design and Discovery (WPI-ICReDD), Hokkaido University, Kita 21 Nishi 10, Kita-ku, 001-0021 Sapporo, Japan

"*Correspondence to [*varnek@unistra.fr*](mailto:varnek@unistra.fr)

**Supporting Information**

1. SMILES/CGR notation
2. Reaction balancing with CGR
3. Examples of reactions discarded by Chemical Filters
4. Simplified reactions generated by autoencoder
5. Comparison of generated and experimentally studied reactions retrieved from SciFinder.

# SMILES/CGR notation

Generally, SMILES/CGR follows the OpenSMILES rules^1^. Unlike regular Daylight SMILES, in OpenSMILES, the ring closure number is given after bond order – it gives an opportunity to easily operate with more than 9 rings and has two-digit ring closure symbols. In the given version of SMILES/CGR, instead of specification of aromatic atoms in lowercase, we used colon for aromatic bonds; the aromatic atoms are given in uppercase. It reduces the diversity of symbols and is much more convenient for the specification of aromatic atoms involved in reaction centers in CGR, especially in cases when aromatic atoms are changed to aliphatic or *vice versa*.

Upon SMILES/CGR generation, the following convention is used: any expression given in squared brackets is considered as one symbol. Thus, two symbol atoms (e.g. [Co]), charged atoms [N+], etc. are considered as special types of atoms. This convention is used by the tokenizer, and it also reduces the complexity of SMILES/CGR generation by autoencoder.

Dynamic bond labels and dynamic atoms are also specified within squared brackets and thus considered as one symbol by tokenizer. Dynamic bonds in CGR have special labels representing changes in bond orders. The list of available dynamic bond labels corresponding to bond order changes is given in Table S1. Dynamic atom corresponds to change of formal charge or radical state of this atom in reaction. Their labels are also given in brackets, including the atom symbol and text keys for atomic property in reactant and product, separated by symbol >. The list of available text keys is given in Table S2. For a neutral atom A gaining a positive charge *+n* in reaction dynamic atom will be encoded as [A0>*+n*]. In the case of charges +1 and -1, the number 1 is omitted. Properties for charges and radicals may be combined consecutively within one pair of brackets, e.g. [A0>-^>*] stands for an atom which becomes an anion-radical.

Table S1. SMILES/CGR text representations for bonds. The corresponding bond type in reactants is shown in rows (From), and the bond type in products is in columns (Into). The main diagonal represents the non-dynamic bond text key for the corresponding bond type.

| **Into**  **From** | **No bond** | **Single bond** | **Double bond** | **Triple bond** | **Aromatic bond** | **Any bond** |
| --- | --- | --- | --- | --- | --- | --- |
| **No bond** | . | [.>-] | [.>=] | [.>#] | [.>:] | [.>~] |
| **Single bond** | [->.] | -^1^ | [->=] | [->#] | [->:] | [->~] |
| **Double bond** | [=>.] | [=>-] | = | [=>#] | [=>:] | [=>~] |
| **Triple bond** | [#>.] | [#>-] | [#>=] | # | [#>:] | [#>:] |
| **Aromatic bond** | [:>.] | [:>-] | [:>=] | [:>#] | : | [:>~] |
| **Any bond** | [~>.] | [~>-] | [~>=] | [~>#] | [~>:] | ~ |

^1^ Usually omitted.

Table S2. Text keys for properties of dynamic atoms in SMILES/CGR.

| **Property** | **Uncharged** | **Positively**  **charged** | **Negatively**  **charged** | **Non-radical** | **Radical** |
| --- | --- | --- | --- | --- | --- |
| **Text key** | 0^1^ | +n | -n | ^ | * |

^1^ Omitted for conventional atoms, used only for dynamic atoms.

SMILES/CGR generation and parsing, including preparation of canonic SMILES/CGR, are implemented into CGRtools Python library^2^. Since generation rules of molecular SMILES represent a subset of the SMILES/CGR rules, the same algorithm was used for SMILES and SMILES/CGR.

1. **Reaction balancing with CGR**

Figure S1. Encoding (*top*) of a reaction into a Condensed Graph of Reaction (CGR) detects the forming and breaking bonds. Subsequent decoding of a CGR back to a chemical reaction equation (*bottom*) balances reactants and product sides by adding the leaving groups, thus, the number of heavy atoms on both sides is equal. Note that since the hydrogens and the solvent in the equation are implicit, vacant valences in the added leaving will be filled with implicit hydrogens, leading to the apparent discrepancy in the number of hydrogens on both sides.

1. **Examples of reactions discarded by Chemical Filters**

Figure S2. Examples of generated reactions, which were discarded by Chemical Filters. They include transformations leading to cleavage of C-C bond (a) or leaving carbon atom (b), and those with unbalanced implicit hydrogens (c and d).

1. **Simplified reactions generated by autoencoder**

Table S3. Simplified reactions with novel reaction centers (new ***RC***) generated by autoencoder. Reactions are grouped according to their ***RC***. Reaction enthalpies were estimated using either bond formation energies (ΔH^bfe^) or in DFT calculations (ΔH^DFT^). ΔH^estim^ is *None* if tabulated values are unavailable.

|  | Structure | ΔH^DFT^, kcal/mol | ΔH^bfe^, kcal/mol |  |
| --- | --- | --- | --- | --- |
|  | **O.BC.NO >> OB.CN.O** | | | |
| 1 | 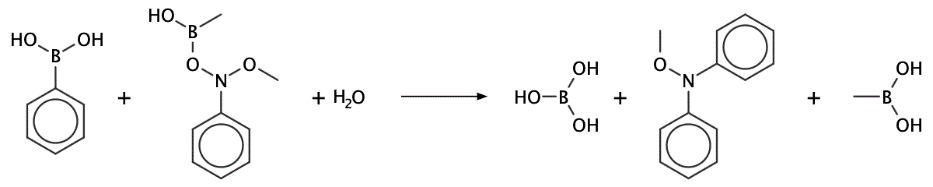 | -56.87 | -67.8 |  |
| 2 | 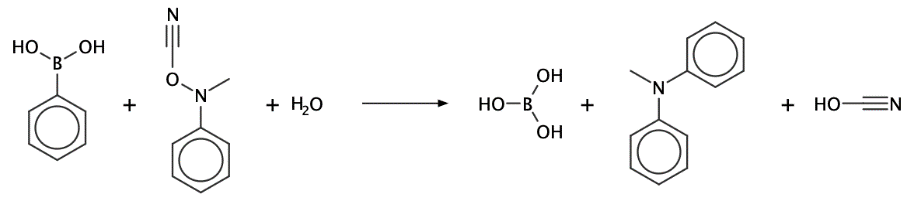 | -27.79 | -67.83 |  |
|  | **O.BC.OI >> OB.CO.I** | | | |
| 3 | 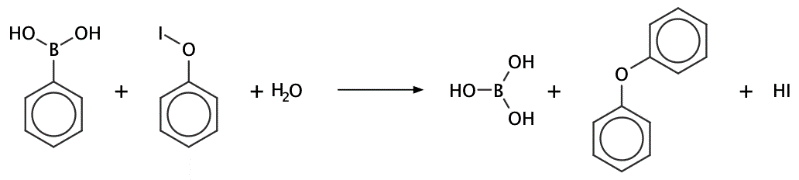 | -44.63 | -41.32 |  |
|  | **O.BC.CS >> OB.CC.S** | | | |
| 4 | 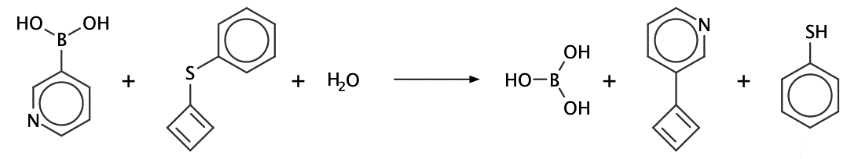 | -30.16 | -37.73 |  |
|  | **O.BC.CN >> OB.CC.N** | | | |
| 5 | 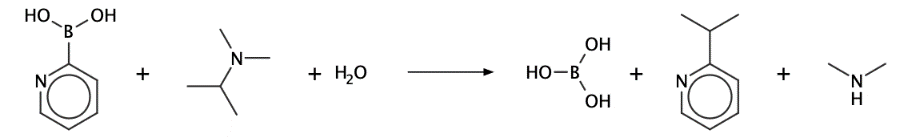 | -38.39 | -35.34 |  |
| 6 | 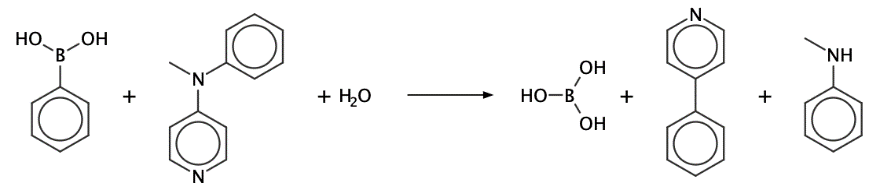 | -32.58 | -35.34 |  |
| 7 | 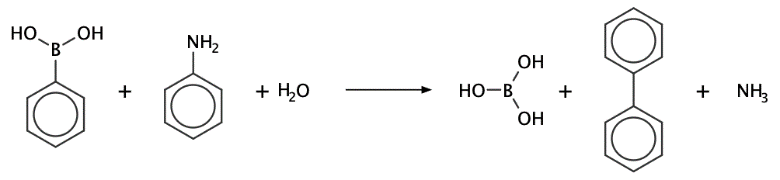 | -31.83 | -35.34 |  |
| 8 | 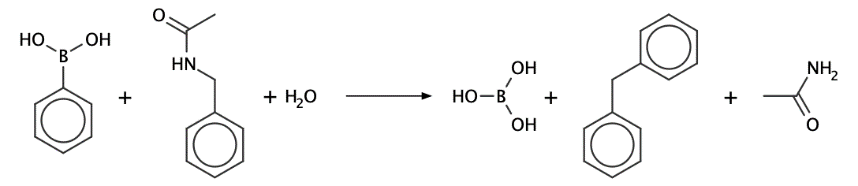 | -31.06 | -35.34 |  |
| 9 | 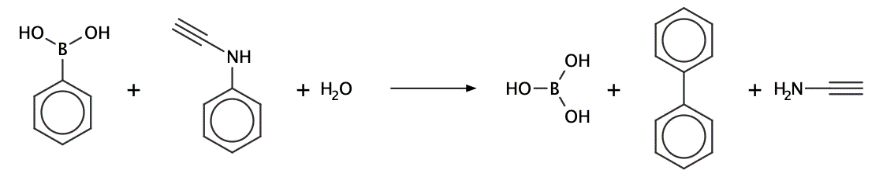 | -30.02 | -35.34 |  |
| 10 | 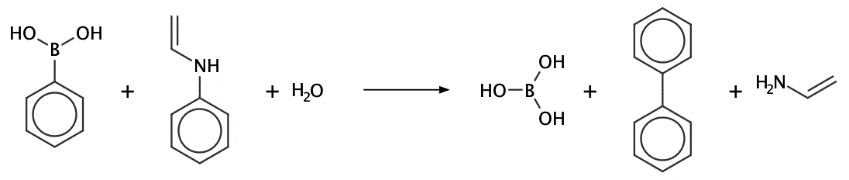 | -28.54 | -35.34 |  |
| 11 | 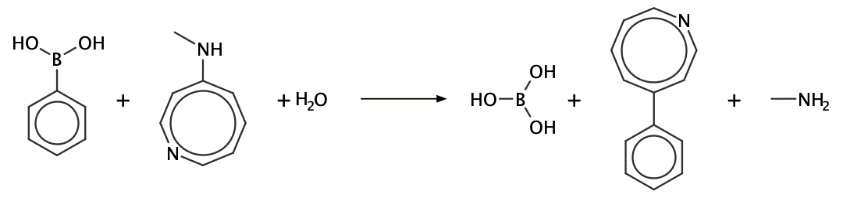 | -22.56 | -35.34 |  |
| 12 | 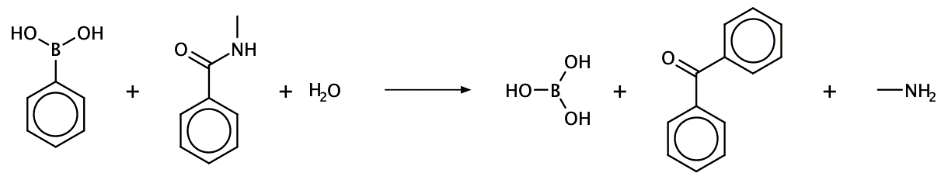 | -12.31 | -35.34 |  |
|  | **O.BC.CF >> OB.CC.F** | | | |
| 13 | 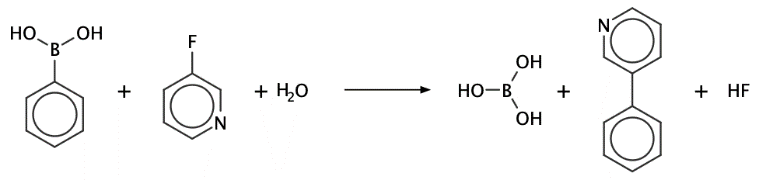 | -36.13 | -35.11 |  |
| 14 | 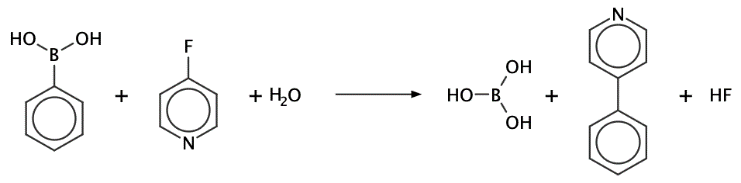 | -34.83 | -35.11 |  |
| 15 | 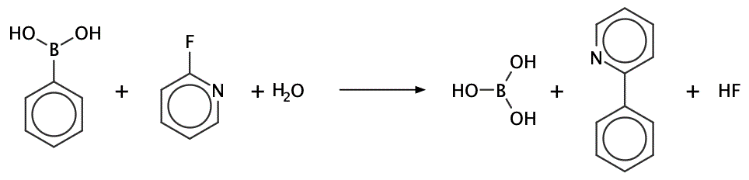 | -31.47 | -35.11 |  |
|  | **O.BC.[Si]S >> OB.C[Si].S** | | | |
| 16 | 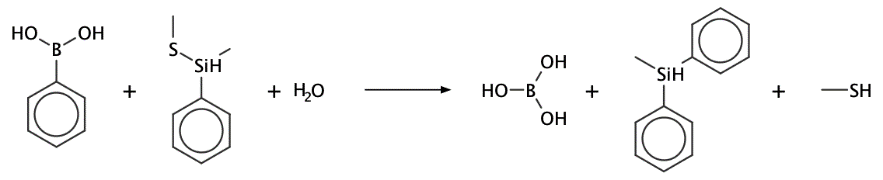 | -9.06 | -26.03 |  |
|  | **O.BC.BrN >> BO.CBr.N** | | | |
| 17 | 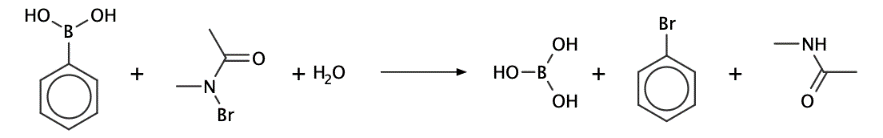 | -53.82 | -22.0 |  |
| 18 | 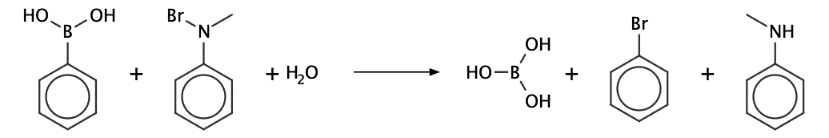 | -49.24 | -22.0 |  |
|  | **O.BC.[Si]Br >> OB.C[Si].Br** | | | |
| 19 | 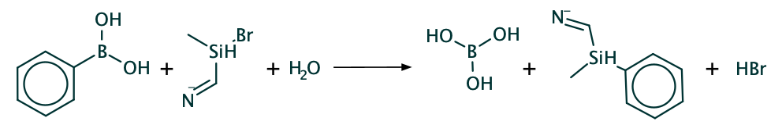 | -10.30 | -21.73 |  |
| 20 | 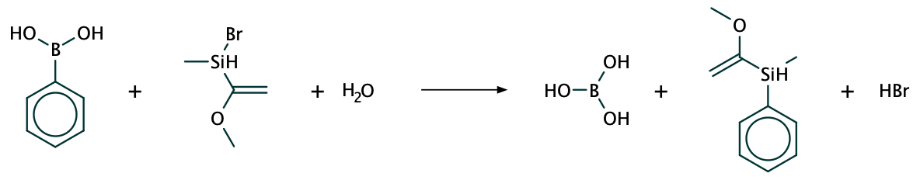 | -1.32 | -21.73 |  |
| 21 | 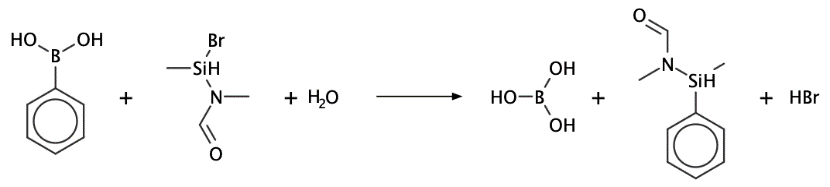 | -1.24 | -21.73 |  |
| 22 | 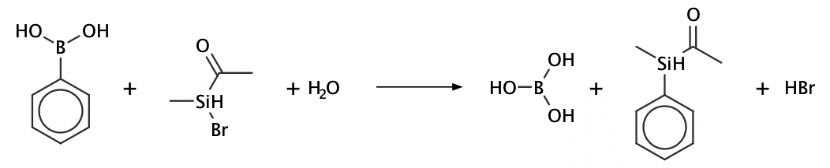 | -0.69 | -21.73 |  |
| 23 | 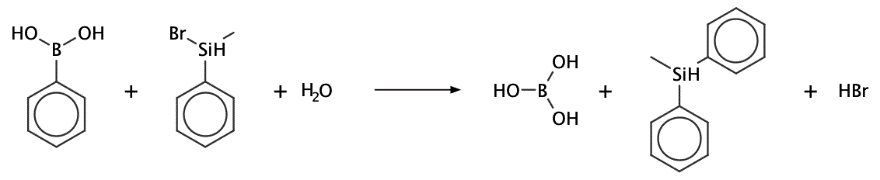 | 0.25 | -21.73 |  |
| 24 | 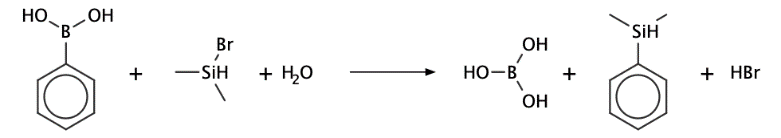 | 0.49 | -21.73 |  |
| 25 | 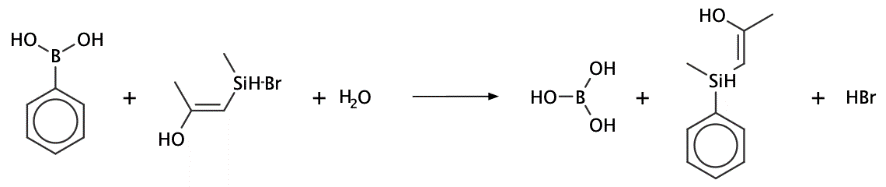 | 0.61 | -21.73 |  |
| 26 | 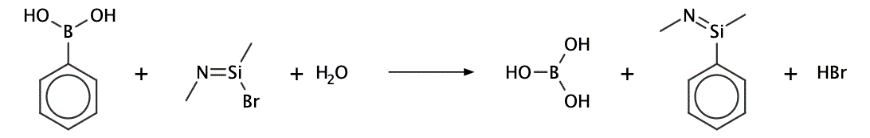 | 0.70 | -21.73 |  |
|  | **O.BC.C[Si] >> OB.CC.[Si]** | | | |
| 27 | 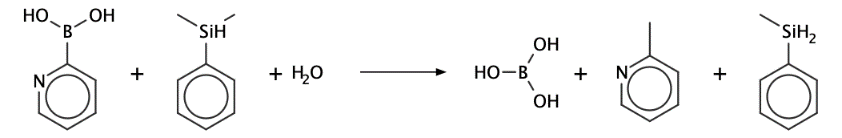 | -24.33 | -16.00 |  |
|  | **O.BC.[Si]O >> OB.C[Si].O** | | | |
| 28 | 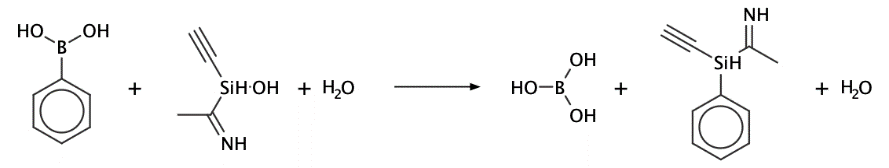 | -1.86 | -10.98 |  |
|  | **O.BC.O[Si] >> OB.CO.[Si]** | | | |
| 29 | 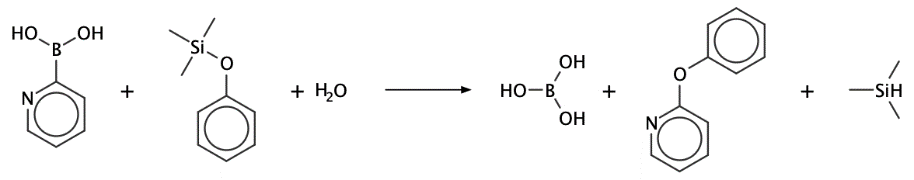 | -2.11 | 13.13 |  |
|  | **O.BC.SBr >> OB.CS.Br** | | | |
| 30 | 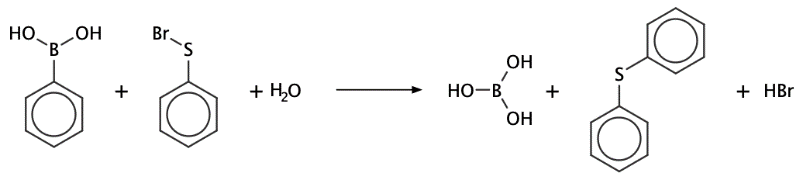 | -27.30 | None |  |
|  | **O.BC.[Si][Sn] >> OB.C[Si].[Sn]** | | | |
| 31 | 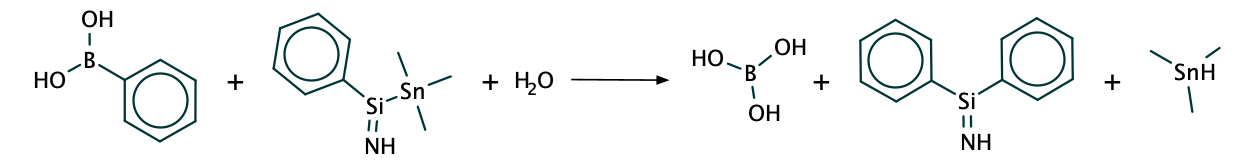 | -16.31 | None |  |

Table S4. Simplified reactions with known reaction center in a new chemical environment (new ***RC+1***) generated by autoencoder. Reactions are grouped according to their ***RC***. Reaction enthalpies were estimated using either bond formation energies (ΔH^bfe^) or in DFT calculations (ΔH^DFT^). ΔH^estim^ is *None* if tabulated values are unavailable.

|  | Structure | ΔH^DFT^, kcal/mol | ΔH^bfe^, kcal/mol |  |
| --- | --- | --- | --- | --- |
|  | **BC.O.CO >> O.CC.BO** | | | |
| 1 | 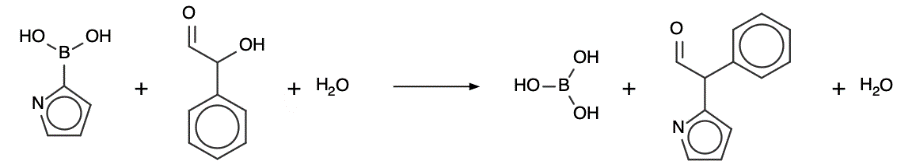 | -65.67 | -40.13 |  |
| 2 | 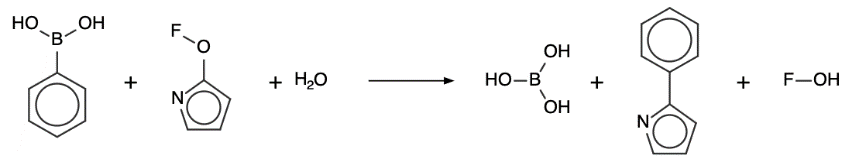 | -56.72 | -40.13 |  |
| 3 | 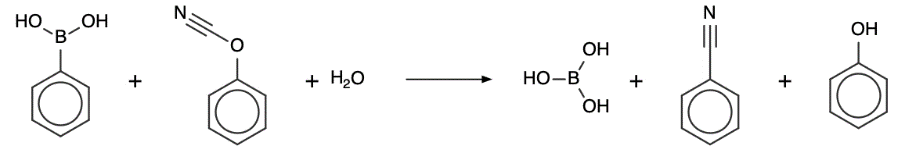 | -49.35 | -40.13 |  |
| 4 | 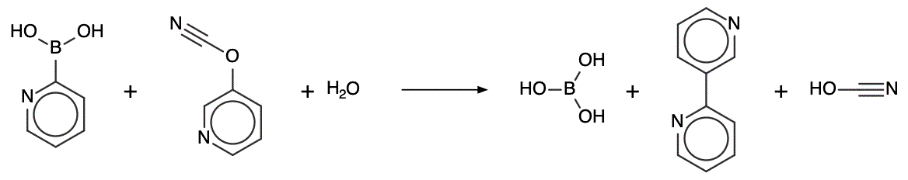 | -43.11 | -40.13 |  |
| 5 | 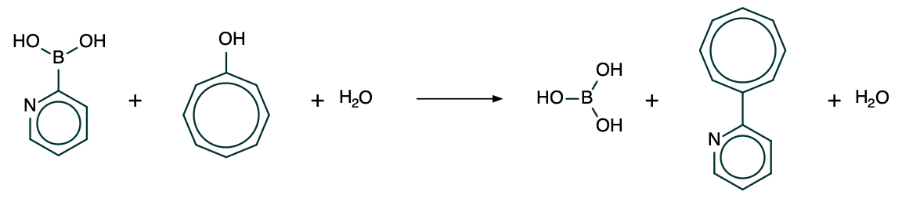 | -38.25 | -40.13 |  |
| 6 | 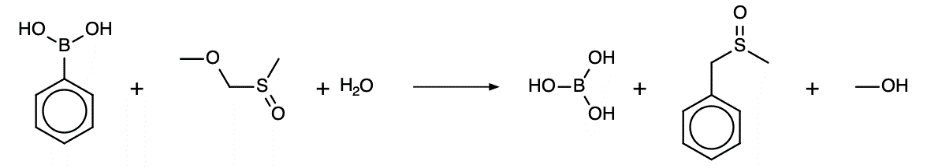 | -37.26 | -40.13 |  |
| 7 | 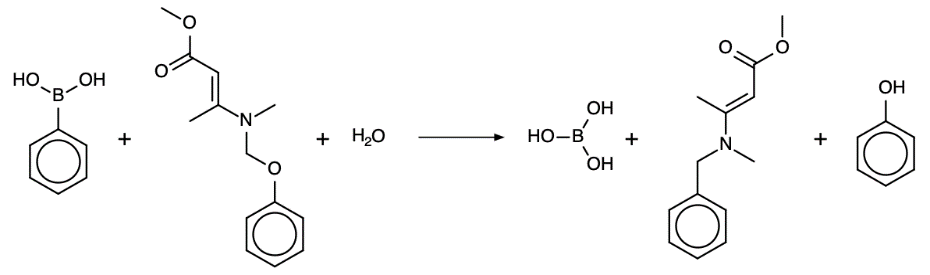 | -34.04 | -40.13 |  |
| 8 | 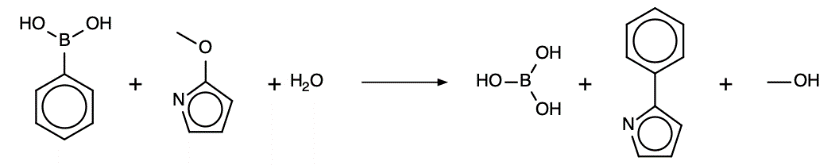 | -31.82 | -40.13 |  |
| 9 | 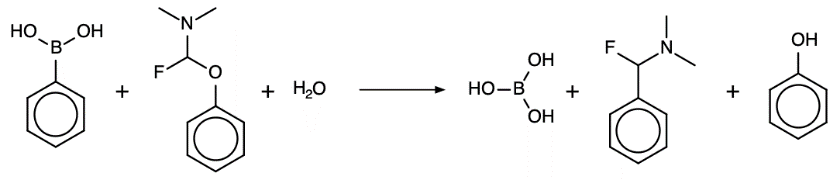 | -27.08 | -40.13 |  |
| 10 | 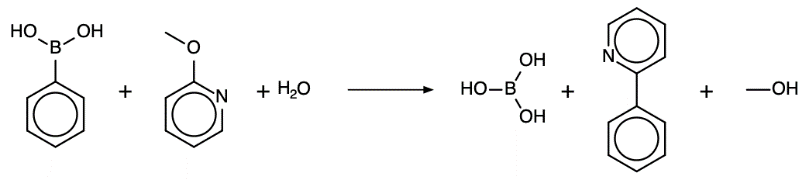 | -25.02 | -40.13 |  |
|  | **BC.O.CI>>I.CC.BO** | | | |
| 11 | 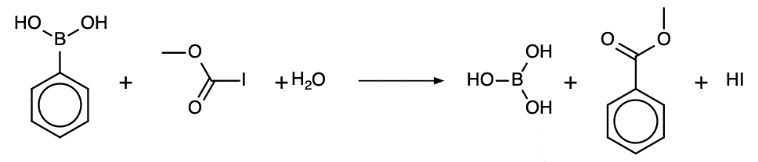 | -31.53 | -35.58 |  |
|  | **BC.O.CBr>>Br.CC.BO** | | | |
| 12 | 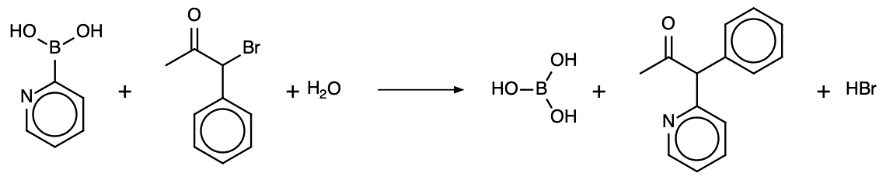 | -34.15 | -34.39 |  |
| 13 | 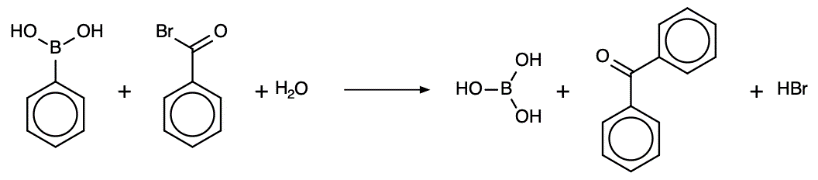 | -21.80 | -34.39 |  |

1. **Comparison of generated and experimentally studied reactions retrieved from SciFinder.**

Table S5. For a given reaction center (new ***RC***), a simplified reaction with novel reaction center generated by autoencoder (top) is compared with its experimentally studied analogue retrieved from SciFinder (bottom). The numeration in the left column corresponds to that in Table S3.

|  | **Reaction center (SMILES)** | **Reaction** | **Ref** |
| --- | --- | --- | --- |
| 5 | **O.BC.CN>>OB.CC.N** |  |  |
|  | Generated reaction | 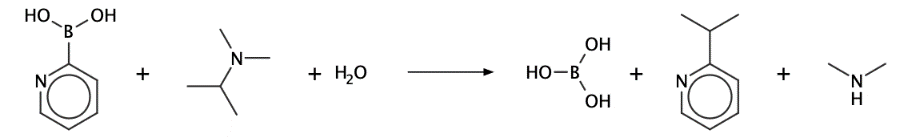 |  |
|  | Reaction in SciFinder | 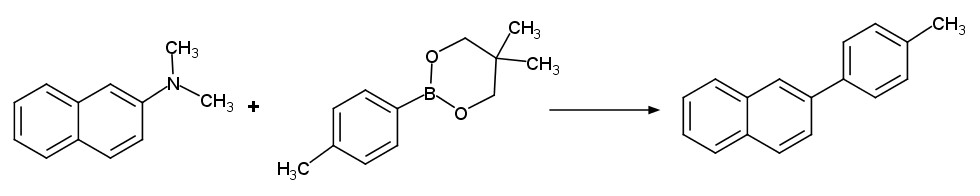 | ^3^ |
| 7 | **O.BC.CN>>OB.CC.N** |  |  |
|  | Generated reaction | 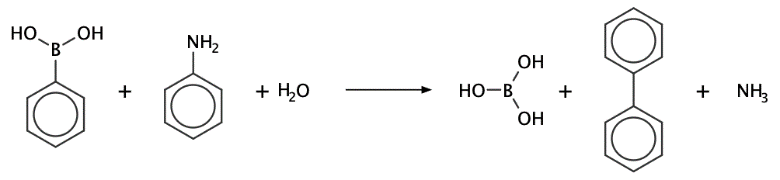 |  |
|  | Reaction in SciFinder | 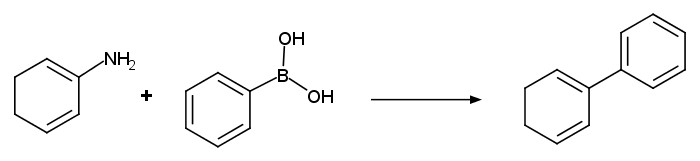 | ^4^ |
| 12 | **O.BC.CN>>OB.CC.N** |  |  |
|  | Generated reaction | 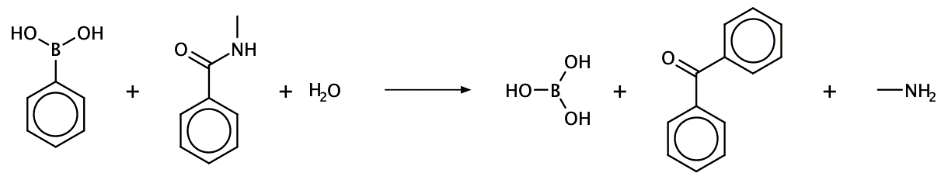 |  |
|  | Reaction in SciFinder | 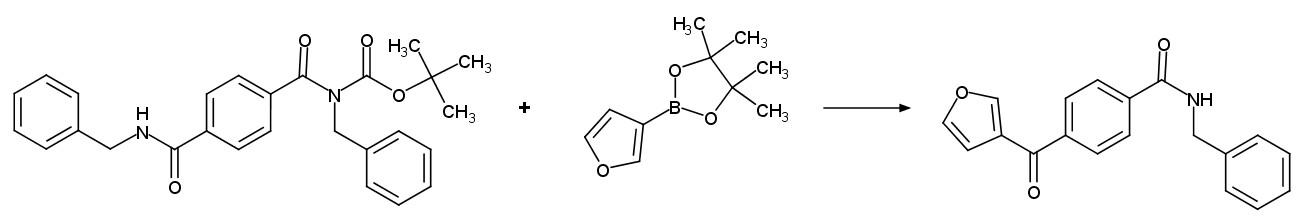 | ^5^ |
| 15 | **O.BC.CF>>OB.CC.F** |  |  |
|  | Generated reaction | 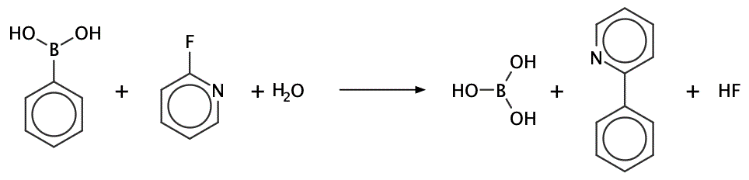 |  |
|  | Reaction in SciFinder | 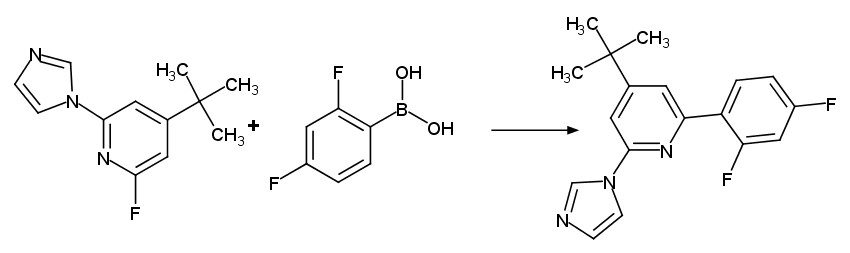 | ^6^ |
| 17 | **O.BC.BrN>>BO.CBr.N** |  |  |
|  | Generated reaction | 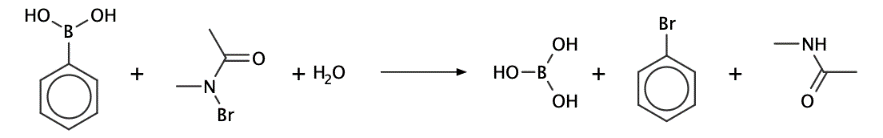 |  |
|  | Reaction in SciFinder | 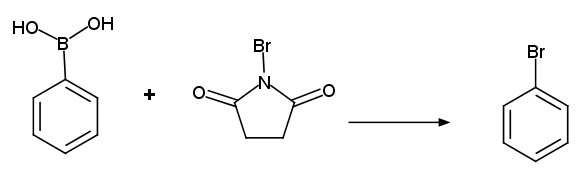 | ^7^ |
| 19 | **O.BC.[Si]Br>>OB.C[Si].Br** |  |  |
|  | Generated reaction | 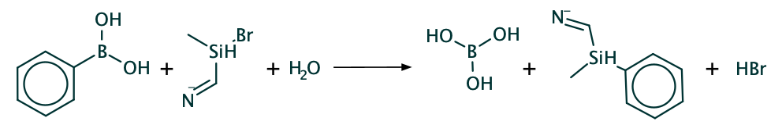 |  |
|  | Reaction in SciFinder | 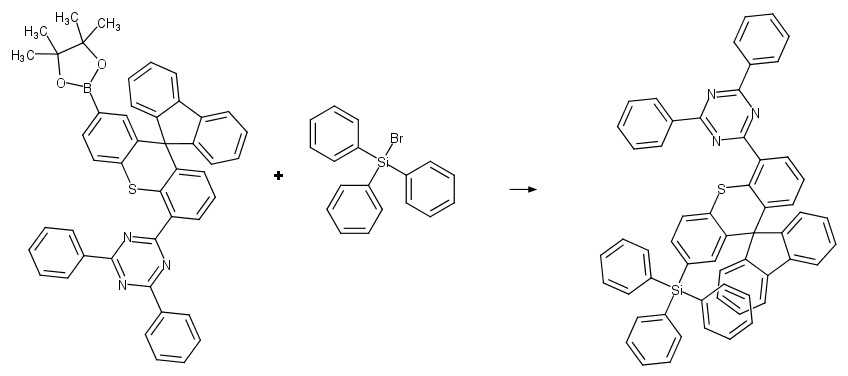 | ^8^ |
| 29 | **O.BC.O[Si] >> OB.CO.[Si]** |  |  |
|  | Generated reaction | 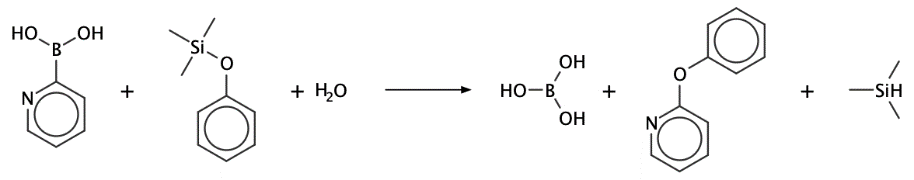 |  |
|  | Reaction in SciFinder | 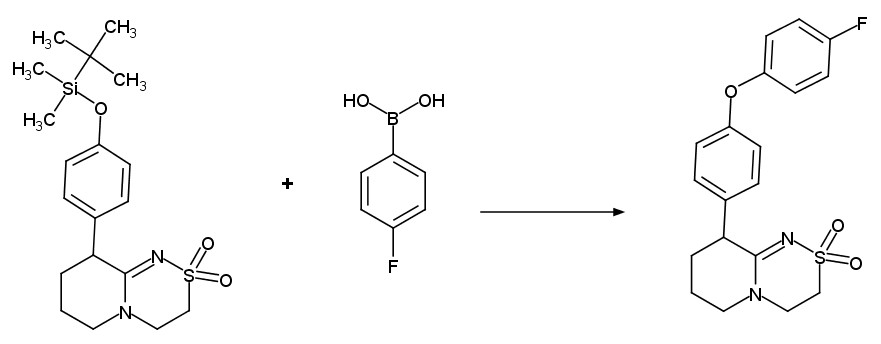 | ^9^ |

Table S6. For a given reaction center in a new chemical environment (new ***RC+1***), a simplified reaction with novel reaction center generated by autoencoder (top) is compared with its experimentally studied analogue retrieved from SciFinder (bottom). The numeration in the left column corresponds to that in Table S4.

|  | **Reaction center (SMILES)** | **Reaction** | **Ref** |
| --- | --- | --- | --- |
| **10** | **BC.O.CO >> O.CC.BO** |  |  |
|  | Generated reaction | 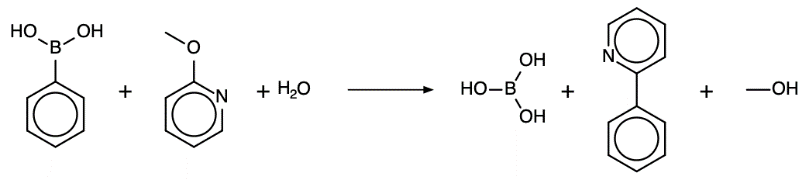 |  |
|  | Reaction in SciFinder | **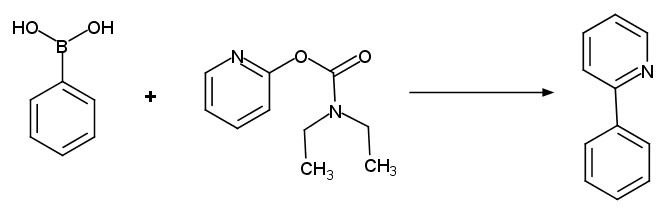** | ^10^ |
| 13 | **BC.O.CBr>>Br.CC.BO** |  |  |
|  | Generated reaction | 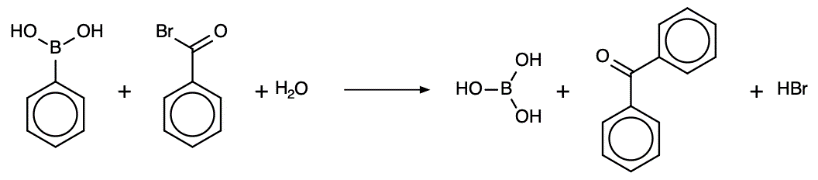 |  |
|  | Reaction in SciFinder | 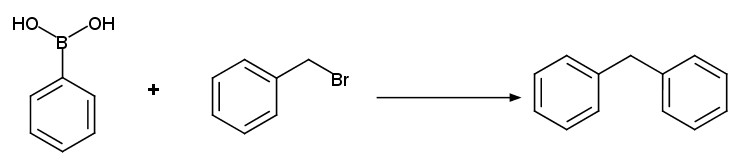 | ^11^ |

**References**

1. James, C. A. OpenSMILES specification. *www.opensmiles.org* (2016).

2. Nugmanov, R. I. *et al.* CGRtools: Python Library for Molecule, Reaction, and Condensed Graph of Reaction Processing. *J. Chem. Inf. Model.* **59**, 2516–2521 (2019).

3. Cao, Z. C., Xie, S. J., Fang, H. & Shi, Z. J. Ni-Catalyzed Cross-Coupling of Dimethyl Aryl Amines with Arylboronic Esters under Reductive Conditions. *J. Am. Chem. Soc.* **140**, 13575–13579 (2018).

4. Zong, Y., Hu, J., Sun, P. & Jiang, X. Synthesis of Biaryl Derivatives via a Magnetic Pd-NPs-Catalyzed One-Pot Diazotization–Cross-Coupling Reaction. *Synlett* **23**, 2393–2396 (2012).

5. Weires, N. A., Baker, E. L. & Garg, N. K. Nickel-catalysed Suzuki–Miyaura coupling of amides. *Nat. Chem.* **8**, 75–79 (2016).

6. Chi, Y. & Lin, J. Iridium complex, OLED using the same, and nitrogen-containing tridentate ligand having carbene unit. *Faming Zhuanli Shenqing* 106928281 (2017).

7. Thiebes, C., Thiebes, C., Prakash, G. K. S., Petasis, N. A. & Olah, G. A. Mild Preparation of Haloarenes by Ipso -Substitution of Arylboronic Acids with N -Halosuccinimides. *Synlett.* 141–142 (1998).

8. Kim, J. J., Hong, W., Yoon, H. & Kang, Y. Preparation of dibenzothiopyrans for organic light-emitting device. *PCT Int. Appl* 2019182411 (2019).

9. Kori, M. *et al.* Fused thiadiazine derivatives as AMPA receptor potentiators and their preparation and use for the treatment of diseases. *PCT Int. Appl.* **16**, 2012020848 (2012).

10. Dindarloo I. I., Majnooni, S., Eslahi, H. & Esmaeilpour, M. Nickel(II) Nanoparticles Immobilized on EDTA-Modified Fe_3_O_4_.SiO_2_ Nanospheres as Efficient and Recyclable Catalysts for Ligand-Free Suzuki–Miyaura Coupling of Aryl Carbamates and Sulfamates. *ACS Omega* **5**, 7406–7417 (2020).

11. Chakraborty, J., Nath, I. & Verpoort, F. Pd-nanoparticle decorated azobenzene based colloidal porous organic polymer for visible and natural sunlight induced Mott-Schottky junction mediated instantaneous Suzuki coupling. *Chem. Eng. J.* **358**, 580–588 (2019).
